# Massive Regime Shifts and High Activity of Heterotrophic Bacteria in an Ice-Covered Lake

**DOI:** 10.1371/journal.pone.0113611

**Published:** 2014-11-24

**Authors:** Mina Bižić-Ionescu, Rudolf Amann, Hans-Peter Grossart

**Affiliations:** 1 Max Planck Institute for Marine Microbiology, Bremen, Germany; 2 Leibniz-Institute of Freshwater Ecology and Inland Fisheries, Stechlin, Germany; 3 Institute for Biochemistry and Biology, Potsdam University, Potsdam, Germany; 4 Berlin-Brandenburg Institute of Advanced Biodiversity Research (BBIB), Berlin, Germany; University of Freiburg, Germany

## Abstract

In winter 2009/10, a sudden under-ice bloom of heterotrophic bacteria occurred in the seasonally ice-covered, temperate, deep, oligotrophic Lake Stechlin (Germany). Extraordinarily high bacterial abundance and biomass were fueled by the breakdown of a massive bloom of *Aphanizomenon flos-aquae* after ice formation. A reduction in light resulting from snow coverage exerted a pronounced physiological stress on the cyanobacteria. Consequently, these were rapidly colonized, leading to a sudden proliferation of attached and subsequently of free-living heterotrophic bacteria. Total bacterial protein production reached 201 µg C L^−1^ d^−1^, ca. five times higher than spring-peak values that year. Fluorescence *in situ* hybridization and denaturing gradient gel electrophoresis at high temporal resolution showed pronounced changes in bacterial community structure coinciding with changes in the physiology of the cyanobacteria. Pyrosequencing of 16S rRNA genes revealed that during breakdown of the cyanobacterial population, the diversity of attached and free-living bacterial communities were reduced to a few dominant families. Some of these were not detectable during the early stages of the cyanobacterial bloom indicating that only specific, well adapted bacterial communities can colonize senescent cyanobacteria. Our study suggests that in winter, unlike commonly postulated, carbon rather than temperature is the limiting factor for bacterial growth. Frequent phytoplankton blooms in ice-covered systems highlight the need for year-round studies of aquatic ecosystems including the winter season to correctly understand element and energy cycling through aquatic food webs, particularly the microbial loop. On a global scale, such knowledge is required to determine climate change induced alterations in carbon budgets in polar and temperate aquatic systems.

## Introduction

On a global scale ice covers, either seasonally or permanently, about 8% of saline and freshwater bodies (not including the Southern hemisphere) [1], [2]. While under-ice phytoplankton blooms are common phenomena (e.g. [3], [4]), little is known about the activity of heterotrophic bacteria during and following such blooms [5]. In the absence of ice coverage, cyanobacterial blooms are known to be accompanied by an increase in bacterial counts and bacterial activity [6]. For example, in four Swedish lakes (two of which had *Aphanizomenon* spp. blooms) bacterial numbers increased during the cyanobacterial bloom to 10^10^ –10^11^ cells L^−1^. At that time the bacterial protein production was between 25 and 350 µg C d^−1^, respectively [6].

It is believed that as a result of low temperatures and reduced nutrient remineralization due to low protozoan grazing, microorganisms under ice are killed, harmed or exist in a dormant state [5], [7], [8]. However, estimated biomass turnover for the overall bacterial communities from these studies usually ranges between 4–10 days indicating that winter microbial communities are actively growing and not just dormant. Although seasonally ice-covered lakes are greatly understudied in the cold season, there is increasing evidence that winter dynamics of the lake's microbiome (prokaryotic and eukaryotic microorganisms) is important for understanding bacterial community succession in the following ice-free period [5], [9].

Powerful molecular tools enable systematic explorations of the diverse and largely uncultivable bacterioplankton communities [10] including the winter season (e.g. [11]). So far, only a few studies [12]–[14] have focused on the bacterial community composition of temporary ice-covered lakes during the winter season. These studies reveal a typical winter bacterioplankton community, which is distinct from that during the warm season indicating specific bacterial adaptation to environmental conditions in winter, e.g. little light, low temperatures and organic matter (OM) concentrations, and high inorganic nutrient availability during winter mixing and early ice cover stages.

Although lakes cover a little more than 3% of the Earth surface as compared to 71% by oceans and seas, they have an equal contribution to CO_2_ emissions [15] and a larger contribution to carbon burial [16]. Therefore, it is surprising that annual studies of freshwater systems often neglect the entire period of ice coverage. Using a well-studied limnic system (Lake Stechlin, Germany), we present evidence for the quantitative significance of heterotrophic bacterial activity due to rapid mineralization of phytoplankton biomass in ice-covered aquatic systems. Our findings thus confirm and extend recent findings on the rapid sinking of under-ice phytoplankton blooms in the Arctic Ocean [4].

In this study we followed a sudden and massive under-ice proliferation of heterotrophic bacteria in oligotrophic Lake Stechlin following the breakdown of an under-ice bloom of the low light-adapted cyanobacterium *Aphanizomenon flos-aquae* in March 2010. Based on this phenomenon during which OM became suddenly available at high concentrations in the cold season, we hypothesize that once winter conditions have reached a steady state, bacterial activity and community composition are mainly regulated by OM availability and subsequent protozoan grazing, and only to a lesser extent by low water temperatures as has been traditionally suggested.

## Materials and Methods

Field permit was granted to the Leibniz institute by the stechlin natural park authorities on a permanent basis.

### Description of study site

Lake Stechlin (53′09 N, 13′02 E) is a seasonally ice-covered meso-oligotrophic freshwater lake formed after the last glacial period in northern Europe (Weichselian). With an area of 4.23 km^2^ and a maximum depth of 69.5 m, this relatively small, lake is one of the deepest lakes of the Northeastern German Lowlands [17]. Similar to other temperate deep lakes, Lake Stechlin overturns in spring and autumn and thus is classified as a dimictic lake [18].

### Sampling procedure

Our main focus was the ice-covered period and the microbial community residing beneath the ice. Samples were collected between 10^th^ December 2009 and 21^st^ April 2010 from the epilimnion (integrated from 0–10 m depth) and the hypolimnion (distinct depth of 20 m) (Table S1). Temperature, oxygen, pH and conductivity were measured with probes (WTW, Weilheim, Germany) to monitor thermal stratification and determine the potential cyanobacterial bloom period. Bacterial protein production (BPP) was determined by [^14^C] - leucine incorporation in triplicates at *in situ* incubation for 1 h as described in Rösel & Grossart [19]. Samples were processed for microscopy, BPP, catalyzed reporter deposition fluorescence *in situ* hybridization (CARD-FISH), denaturing gradient gel electrophoresis (DGGE) and 454 tag pyrosequencing.

For CARD-FISH, water samples were fixed for 24 h at 4°C with formaldehyde (1% final concentration). Subsequently, aliquots were separated to determine particle-attached (PA) and free-living (FL) bacteria by sequential filtration through polycarbonate filters (5 and 0.2 µm pore size, respectively [Millipore, Eschborn, Germany]) as previously described in Rösel et al. [12]. CARD-FISH was done according to Pernthaler et al. [20] with minor modifications as described in Pizzetti et al. [21]. All probes used in this study are given in Table S2. Automated image acquisition and analysis was done as previously described in Zeder et al. [22]. Shortly, analysis of samples of FL bacteria was performed in a fully automated manner using a multi-purpose imaging system (MPISYS, modified after Zeder et al. [22] based on a fully motorized epifluorescence microscope (AxioImager.Z2m, Zeiss, Jena, Germany). To analyze PA bacteria the MPISYS was ran in a semi-automated mode as the density of particles varied from sample to sample. Upon selection, the system acquired a z-stack of 25 images around the focused position with an interval of 0.3 µm for each channel. For cell quantification, a maximum intensity projection was calculated on each channel. Following acquisition FL and PA sample images were analyzed using ACMEtool2 (www.technobiology.ch).

Samples for DNA extraction were size-fractionated immediately after sampling and stored at −80°C for further processing. DNA from 20 epilimnic and 20 hypolimnic samples (10 PA and 10 FL per strata) (Table S1) was extracted as described by Ionescu et al. [23]. To reveal the temporal resolution of the bacterial community composition (BCC), a segment of the 16S rRNA gene was amplified from the DNA extracts using the primer set 341F – GC and 907R [24], after which the amplicons were analyzed by DGGE as detailed in Rösel et al. [12]. The DGGE gels were analyzed using the Phoretix 1D version 11.4 (TotalLab) software and cluster analysis was done with PAST3 [25] using the Bray-Curtis similarity measure. Samples for 454 tag pyrosequencing were then selected based on changes in BCC during the ice-coverage detected by DGGE analysis. A total of 32 epilimnic and hypolimnic samples of PA and FL bacteria were pooled into 8 representative samples (Table S1). 454 tag pyrosequencing for bacterial diversity, using primer sets 28F and 519R [26], was done by MrDNA (Shallowater, Texas), using a Roche 454 FLX Genome Sequencer system equipped with titanium technology.

### Sequence Analysis

Sequences were analyzed as previously described by Ionescu et al. [23], using the bioinformatics pipeline of the SILVA rRNA gene database project and the SILVA SSURef dataset (release 111; [27]). The SILVA databases include all high quality published sequences regardless of their marine, limnic or terrestrial origin. Furthermore, it follows the approved list of bacterial species (www.bacterio.net) and the bacterial code of nomenclature; therefore, differences in nomenclature may occur when compared to uncurated databases. Diversity and community structure analyses were performed based on >132,000 bacterial sequences. A detailed summary of the analysis process of the pyrosequencing data of each sample, including the total number of reads and length distribution, as well as the results of quality management, dereplication and clustering, can be found in the supplement (Table S3). A detailed list of the final taxonomic affiliation of all analyzed sequences together with their relative abundances within the amplicon pool is given in Table (S4) (Bacteria). The sequence data presented in this study can be freely obtained from the European Bioinformatics Institute (Study number PRJEB5107).

## Results

### Environmental conditions

**Figure 1:**
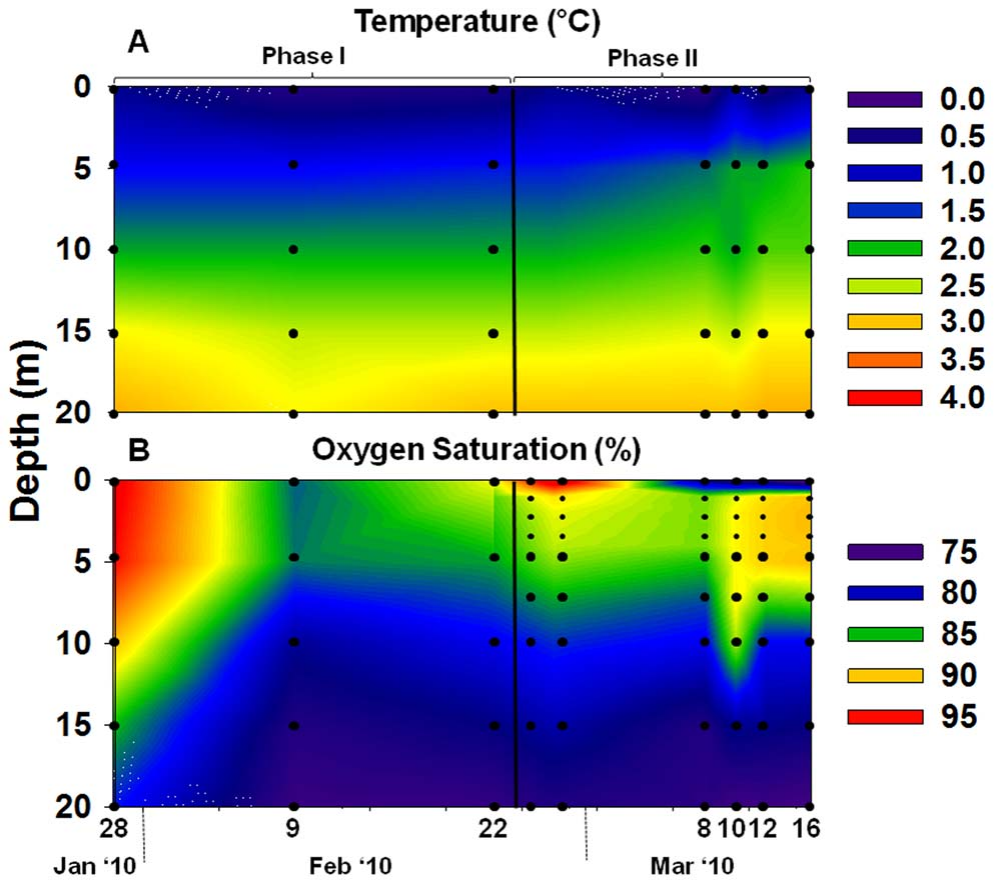
Temperature (A), and oxygen saturation (B) profiles during Phase I and Phase II of the bloom. Black circles represent sampling points. Data are available at a higher spatial resolution only during Phase II of the bloom (small black circles).

In winter 2009/10, ice on the surface of Lake Stechlin first appeared at the end of December. Throughout January, the lake was covered by a thickening layer of clear ice. The ice (∼30 cm thick) remained clear until mid-February, but thereafter the ice sheet was covered by >50 cm of snow. Thermal stratification under the ice was inverted as compared to the summer stratification. In the upper 5 m the water was <1°C while between 5 and 20 m the temperature increased to 3.1°C (Fig. 1A). Below 20 m the temperature increased gradually, but remained below 4°C. The ice-off occurred in the second half of March.

Oxygen was overall below 100% saturation (450 and 405 µmol L^−1^ in surface and bottom water, respectively) throughout the ice-coverage period with a background of 75% saturation (Fig. 1B, S1A). Until the end of February 2010, O_2_ saturation level in the upper 4–5 m increased to maximal 95% following a surface bloom of the low light-adapted cyanobacterium *Aphanizomenon flos-aquae* [28]. Thereafter, the O_2_ saturation levels in the same water stratum decreased to 75% in early March (Fig. 1B) when the ice was covered by a thick layer of snow. The increase and decrease in O_2_ saturation levels during February to March 2010 are accompanied by a respective increase and decrease in pH values (Fig. S1B).

### Cyanobacteria dynamics

Two cyanobacterial blooms were observed in Lake Stechlin in 2009, during September (*A. flos*-*aquae* and *Anabaena macrospora*) and December (*A. flos*-*aquae*), respectively [28]. The second bloom, initiated by the mixing of the water column in late fall, started in November and peaked during December. Although *A. flos-aquae* in Lake Stechlin forms heterocysts in summer, no heterocysts were visible in any of the analyzed filaments collected in winter (n>200 filaments), consistent with data on available combined nitrogen (20 µmol L^−1^ NO_3_; [28]). The second bloom persisted during the clear-ice period of the lake (December to mid-February). As the ice became covered by snow reducing the incident photosynthetically active radiation to 40 and 5 µmol photon m^−2^ s^−1^ at 1 and 5 m, respectively [28]. Due to the combination of low light and low temperature, the cyanobacteria became physiologically stressed [28] and a rapid bloom (>3 fold in 8 days) of heterotrophic particle-attached (PA) and subsequently free-living (FL) bacteria occurred.

**Figure 2:**
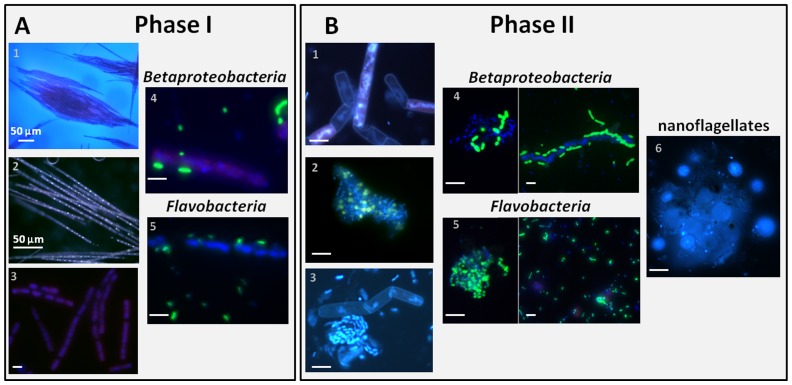
Micrographs of DAPI and FISH stained *Aphanizomenon flos*-*aquae* filaments and heterotrophic bacteria, respectively; collected from the epilimnion during Phase I (A) and Phase II (B). Cyanobacteria in Phase I are found in tufts (A1), the filaments contain poly-P granules (A2) and are hardly colonized by heterotrophic bacteria (A3). In Phase II single filaments with ghost cells are visible (B1). These are often aggregated with poly-P granules spilled onto the aggregate (B2) and heavily colonized by heterotrophic bacteria (B3). Significant increase in colonization between *Betaproteobacteria* and *Flavobacteria* in Phase I (A4–5) and Phase II (B4–5) is visible. End of Phase II is characterized by an increase in nanoflagellates (B6).

Therefore, we divided the sampling period during lake ice coverage into two phases. Phase I (January 28^th^-February 22^nd^) comprises the late bloom of *A. flos-aquae*. Phase II (February 23^rd^-March 16^th^) depicts the breakdown of the cyanobacterial bloom and the rapid rise of heterotrophic bacteria. Differences between Phases I and II are evident from micrographs of DAPI and FISH stained cyanobacterial filaments collected from the epilimnion (Fig. 2). First, the bloom of *A. flos-aquae* consists of intact filaments that contain poly-P granules and are rarely colonized by heterotrophic bacteria (Fig. 2A, S2). Subsequently, in Phase II damaged filaments with “ghost cells” are often aggregated and the poly-P granules are no longer associated with the filaments. These aggregates are heavily colonized by heterotrophic bacteria (Fig. 2B, S2).

**Figure 3:**
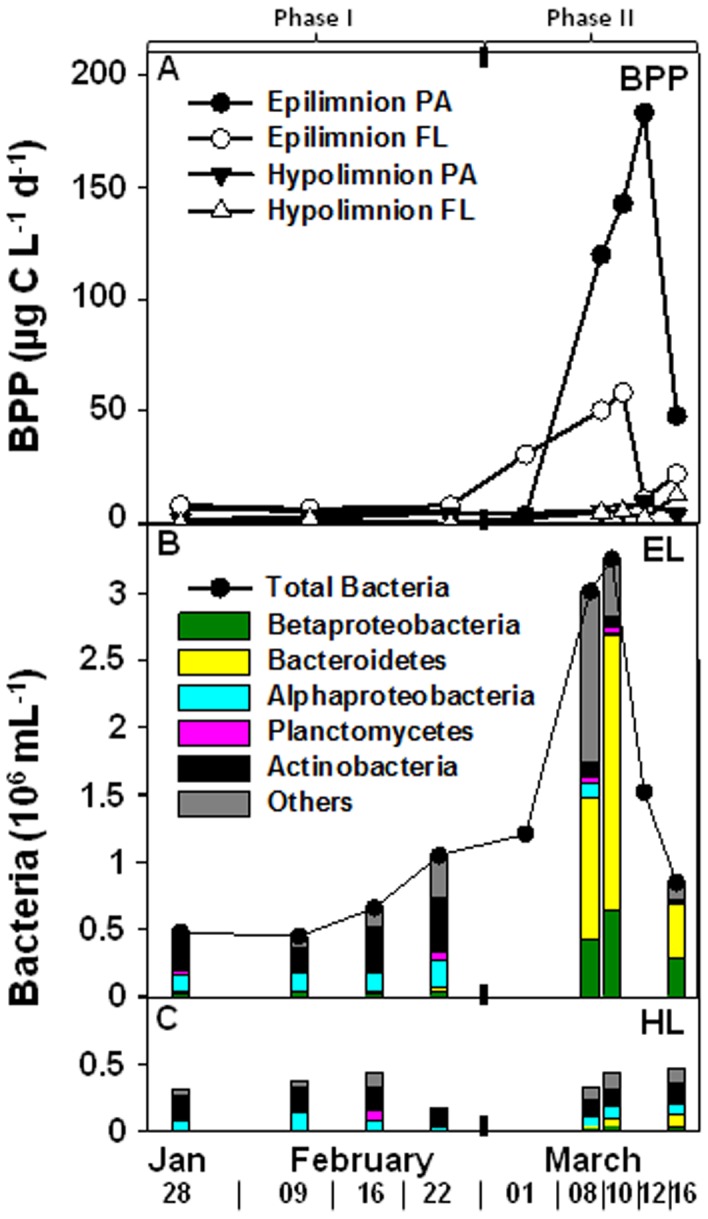
Bacterial protein production (BPP) of particle-attached (PA) and free-living (FL) bacteria in the epilimnion and hypolimnion (A) and bacterial abundance of total bacteria in the epilimnion (B) and hypolimnion (C) during the two phases of the bloom.

In the epilimnion (0–10 m), BPP of PA and FL bacteria, increased abruptly from Phase I to Phase II (5 to 183 and 8 to 58 µg C L^−1^ d^−1^, respectively) (Fig. 3A). This indicates that BPP can be very high at temperatures <1°C as compared to the rest of the year in Lake Stechlin or to other aquatic ecosystems [29]. The increase in BPP in Phase II was first seen in the FL fraction, but was followed by a larger increase in BPP of the PA fraction. The subsequent breakdown of the *A. flos-aquae* bloom led to a sudden rise in abundance of heterotrophic bacteria from 1×10^6^ to 3.25×10^6^ cells mL^−1^ (Fig. 3B). At the end of Phase II, BPP in the PA and FL fraction rapidly decreased to 48 and 22 µg C L^−1^ d^−1^, respectively; still several folds higher than at the end of January when the lake surface was covered by clear ice. BPP eventually decreased as the number of bacterial cells was reduced to 0.85×10^6^ cells mL^−1^, parallel to the increase in abundance of heterotrophic flagellates.

**Figure 4:**
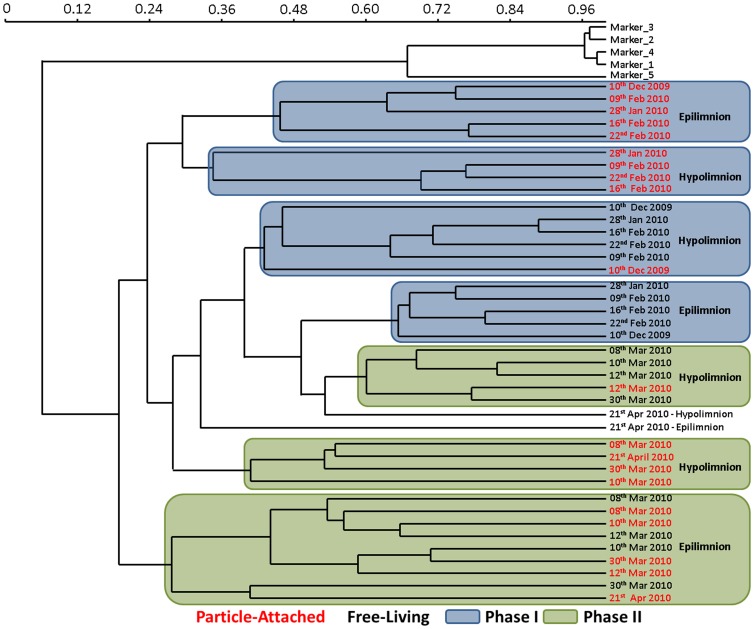
Denaturing gradient gel electrophoresis dendrogram showing the relationship between particle-attached and free-living bacterial communities from epilimnion and hypolimnion of Lake Stechlin during Phase I and Phase II of the bloom. The gels were analyzed using the Phoretix 1D version 11.4 (TotalLab) software and cluster analysis was done with PAST3 [25] using the Bray-Curtis similarity measure.

**Figure 5:**
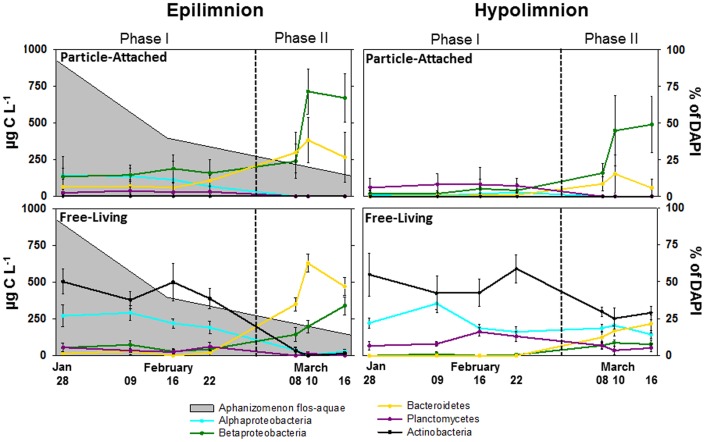
Distribution of relative abundances of major phylogenetic groups (% of DAPI - right axis): *Alphaproteobacteria*, *Betaproteobacteria*, *Bacteroidetes*, *Planctomycetes* and *Actinobacteria* as assessed by CARD-FISH in the particle-attached and free-living fractions in both epilimnion and hypolimnion. Phase I and II of the bloom are separated by the dashed line. The abundance of *Aphanizomenon flos*-*aquae* (gray field) is presented during both phases of the bloom (left axis).

**Figure 6:**
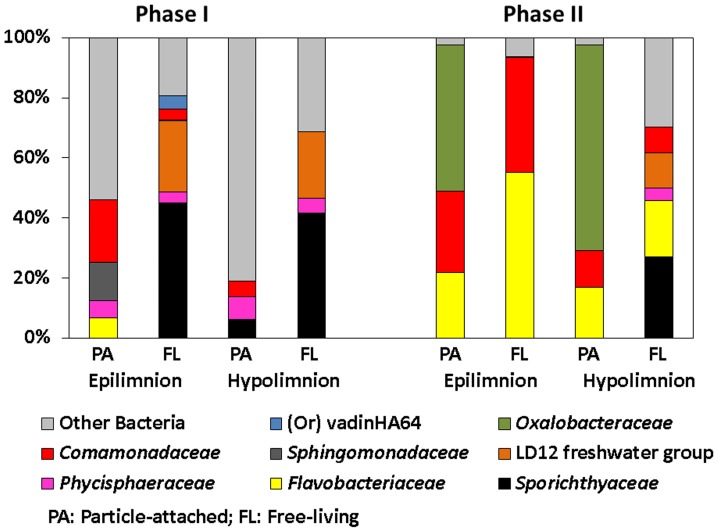
Sequence frequencies of major particle-attached (PA) and free-living (FL) bacterial families comprising to >5% of total sequences in at least one sample in the epilimnion and hypolimnion of Lake Stechlin during Phase I and Phase II of the under-ice bloom.

The sudden increase in bacterial abundance and activity were accompanied by a similarly rapid shift in Bacterial Community Composition (BCC) as evident from Denaturing Gradient Gel Electrophoresis (DGGE), Catalyzed Reporter Deposition Fluorescence *In Situ* Hybridization (CARD-FISH) and 454 tag pyrosequencing results (Fig. 4 & S3; 3B & 5; 6 & S4 respectively). The swiftness of the change is best observed by high temporal resolution DGGE fingerprinting, showing the presence of two, stable, yet different communities in Phase I and II of the bloom (Fig. 4; Fig. S3). In Phase I, members of *Alphaproteobacteria*, *Betaproteobacteria, Bacteroidetes* and *Planctomycetes* (11% (0–40%), 16% (0–33%), 7% (0–19%) and 3% (0–28%), respectively) were associated with *A. flos-aquae* cells (Fig. 5). In Phase II, we observed a simultaneously strong increase in relative abundances of *Betaproteobacteria* and *Bacteroidetes* (54% (4–88%) and 31% (4–63%), respectively). These were also the only two bacterial groups associated with *A. flos-aquae* at that time. *Alphaproteobacteria* and *Actinobacteria* (on average 24% and 44%, respectively) dominated the FL fraction in Phase I, but were replaced in Phase II by a dominance of *Bacteroidetes* and *Betaproteobacteria* (on average 49% and 23%, respectively) (Fig. 3B; 5). Comparative 16S rRNA gene sequencing of pooled PA and FL fractions of both the epi- and hypolimnion showed that the transition between Phase I and II resulted not only from a change in the bacterial groups, as already implied by CARD-FISH (Fig. 3B; 4), but also from a strong decrease in the number of dominant bacterial families, and the expansion of a few groups of specialist bacteria (Fig. 6 & S4). In Phase I, 50% of PA bacteria in the epilimnion belonged to 4 major families while the rest was distributed among 44 others. In contrast, in Phase II, 96% of the PA bacteria were dominated by 3 families alone: *Flavobacteriaceae* (21%), *Comamonadaceae* (27%) and *Oxalobacteraceae* (48%). While the first two families dominated the PA fraction in the epilimnion during Phase I, *Oxalobacteraceae* contributed to <0.5% of the BCC (Table S4). During the transition from Phase I to Phase II, FL bacteria in the epilimnion showed a similar succession as PA bacteria resulting in a decreased bacterial diversity. These changes in PA and FL BCC did not occur simultaneously. A succession between the PA and FL fractions is obvious from the DGGE fingerprints, as several bands appeared first on particles and only later in the surrounding water (Fig. S3). Cluster analyses further shows the strong link between the PA and FL fractions in Phase II of the bloom where in contrast to Phase I PA and FL communities cluster together (Fig. 4).

Our results indicate that the hypolimnion (20 m) was affected by the events in the epilimnion only towards the end of Phase II. Total bacterial numbers (FL) in the hypolimnion (20 m) were <0.5×10^6^ cells mL^−1^ throughout the entire period. Accordingly, BPP was much lower than in the epilimnion (1–9 and 0.1–12 µg C L^−1^ d^−1^ in PA and FL, respectively) during the whole period and only slightly increased at the end of Phase II (Fig. 3A). This, together with our microscopic observations, indicates that solely a small fraction of cyanobacterial cell debris reached this depth, and that the cyanobacterial biomass was efficiently remineralized in the epilimnion.

## Discussion

In temperate lakes, persistent cyanobacterial blooms frequently occur in winter under the ice (e.g. [30]) (Table S5). However, most ice-covered (meso-) oligotrophic lakes such as Lake Stechlin have been considered to be low carbon environments in winter [5]. This is mainly because of two factors: 1) Primary productivity is usually low due to decreased light availability under the ice; 2) Input of atmospheric and terrestrial OM is blocked by the ice coverage and the available internal OM is rapidly consumed by the residual heterotrophic bacterial community. Accordingly, a low BPP was measured during Phase I of the cyanobacterial bloom. Coverage of Lake Stechlin with clear ice, however, allowed for penetration of sufficient light which together with a stable inverse (as compared to the summer) stratification of the water column sustained growth of low temperature- and low light-adapted *A. flos-aquae* [28] in the cold epilimnion (<4°C).

Bacterial numbers and BPP remained low in Lake Stechlin in Phase I. At this time inorganic nutrients necessary for the blooming of *A. flos-aquae* were abundant after the mixing in early winter, including nitrogen as indicated by the lack of heterocysts and phosphorus by storage in form of poly-P granules. The high growth potential of the winter bacterioplankton - irrespective of temperature - became evident when *A. flos-aquae* became light limited [28] due to low irradiance under the snow-covered ice in Phase II. The sudden increase in bacterial abundance and BPP in the epilimnion during the transition from Phase I to Phase II in parallel to the relatively high availability of inorganic nutrients support the hypothesis that the winter bacterioplankton in Lake Stechlin is controlled by carbon availability rather than temperature. The physiologically stressed cyanobacteria were rapidly colonized by heterotrophic bacteria that alone, or in combination with cyanophages degraded the *A. flos-aquae* filaments, leading to an extremely high bacterial abundance and BPP in Phase II. The degradation of cyanobacterial filaments resulted in the release of the protoplasmic content into the surrounding water leaving empty “ghost cells” (Fig. 2B), thus increasing OM availability also for FL bacteria. Although temperature remained <4°C in the epilimnion in Phase II, high inorganic nutrient availability coupled to the relief from carbon limitation led to an extreme increase in BPP, which was >5 times higher than measured during the peak of the subsequent spring bloom [19]. Moreover, BPP under the ice is in the same range as measured during summer blooms in four Swedish lakes despite the fact that the bacterial abundance under the ice was 1–2 orders of magnitude lower [6]. From this notion it is evident that periodical events such as the under ice *A. flos-aquae* bloom in Lake Stechlin lead to high microbial OM turnover in winter when concentrations of herbivorous zooplankton are low [5]. This suggests that availability of OM rather than temperature triggered BPP leading to a rapid mineralization of the phytoplankton biomass in ice-covered Lake Stechlin counter-acting its sinking and burial in the sediment. Thus, the rapid sinking of under-ice phytoplankton blooms in cold environments [4] should be seen in an environmental context and should not be considered as a general rule.

It can be assumed that the high heterotrophic activities measured in Phase II have the potential to strongly affect C cycling, particularly the release of CO_2_ from the lake into the atmosphere after ice-off. CO_2_ concentrations under the ice gas-barrier can exceed summer values [31] reaching concentrations of up to 650 µmol L^−1^ and often 2–3 folds higher than during the ice-free period [32]. Hence, the resultant CO_2_ buildup can be rapidly released to the atmosphere upon ice-off. So far, under ice blooms of heterotrophic bacteria have been neglected for modeling of metabolism and element cycling in lakes. However, Karlsson et al. [33] suggest that up to 55% of the annual gas emission from freshwater systems occurs during ice-off. To better understand and predict environmental consequences of lake biogeochemistry, year-round studies are required to also include massive under ice blooms of photoautotrophic and heterotrophic microorganisms. These may also set the frame for the subsequent food web development during the growing season [5].

High availability of OM during bacterial colonization and lysis of *A. flos-aquae* cells also dramatically affected biodiversity reflected by a temporal shift in BCC. Our DGGE fingerprints suggest a sudden change of BCC between Phase I and II. The uniformity in quality of the available OM in Phase II of the bloom was indicated by the dominance of solely a small group of specialized bacteria different from those in Phase I. These bacteria first appeared in the epilimnic PA fraction and later in the FL fraction of the epilimnion and in the PA fraction of the hypolimnion. This suggests that a) bacterial colonization of senescent cyanobacteria resulted in detachment of bacteria and release of substrates into the surrounding water supporting the proliferation of these communities; b) a small fraction of colonized *A. flos-aquae* aggregates sank into the hypolimnion. The latter process was reflected by significant changes in BCC of hypolimnic PA bacteria greatly reducing microbial diversity in this fraction.

These shifts in microbial communities present a limnic under-ice analogue to the substrate-driven succession of marine bacterioplankton populations induced by phytoplankton blooms recently described by Teeling et al. [34]. Although temperatures and salt content greatly differed between the two studies, the *Flavobacteriaceae* family, known to degrade high-molecular-weight OM [35], were among the first to colonize the *A. flos*-*aquae* cells. Nevertheless, while the marine event harbored numerous genera from the *Flavobacteriaceae*, *Flavobacterium* was the sole genus detected under the ice in Lake Stechlin (Fig. S4). In the marine analogue *Gammaproteobacteria* and to some extent *Alphaproteobacteria* succeeded the *Flavobacteriaceae*. In contrast, in our study, *Betaproteobacteria* (Fig. 6), specifically members of the genera *Undibacterium* and *Massilia* as well as an unclassified group of *Comamonadaceae* (Fig. S4) followed the initial colonization and rapidly exceeded the abundance of *Flavobacterium* (Fig. 5). We propose that the betaproteobacterial clades detected under the ice in Lake Stechlin fill in similar ecological niches in freshwater as the marine *Gammaproteobacteria* associated with a decaying algal bloom in Teeling et al. [34]. Interestingly, both blooms independent of water temperature had a similar carrying capacity, leading to an increase in FL bacterial abundance from 0.5×10^6^ to 3×10^6^ cells mL^−1^. This maximal carrying capacity could be a result of a protozoan top-down control similar to that observed in Lake Stechlin.

In our study, available OM affects the BCC and BPP. While the temperature in the epilimnion remains constant <1°C throughout the period, and, therefore, can be excluded as an influencing factor. OM increases dramatically in Phase II due to cyanobacterial decay. Based on our DGGE results, the BCC remained stable during Phase I (for at least 2 months) in parallel to a rather stable BPP. Nevertheless, during the rapid transition between Phase I and II of the bloom, while temperature remained constant, we observed a major change in BCC accompanied by a large increase in activity. The initially low abundant *Flavobacteriaceae* in Phase I appeared in larger numbers in Phase II and the previously diverse *Comamonadaceae* were reduced to 3 genera solely (Fig. S4). These phylotypes included cold adapted species such as *Flavobacterium psychrolimnae*, *F. fryxellicola*, *F. limicola*, *F. omnivorum*, *F. frigidarium* that are often reported from ice-covered aquatic systems [36]–[39]. The bacterial phylotypes in Phase II grew rapidly and were able to degrade the cyanobacterial cells to a large extent. This suggests that availability of OM not only triggered BPP, but also succession in BCC.

Under-ice blooms of phytoplankton are not sporadic events since, during the last decades, they have been regularly described from limnic environments and more recently also from marine systems (Table S5). Using Lake Stechlin as an example, we show that temperate lakes bear the potential for high biological activity even during the cold winter season. Despite the low temperature (<4°C), carbon flow from primary production and, particularly, from cyanobacteria biomass during bloom breakdown has the potential to fuel massive blooms of heterotrophic microorganisms. These were soon top-down controlled in the epilimnion by heterotrophic nanoflagellates (Fig. 2B), demonstrating that also protozoa growth was less dependent on water temperatures than on prey availability. Consequently, under ice blooms of photoautotrophic and heterotrophic microorganisms greatly affect the lake's biogeochemistry and thus set the frame for further food web dynamics in the ice-free season.

Current climate change leads to a reduced intensity of ice coverage and hence to dramatic environmental changes with so far unknown ecological consequences, e.g., for biogeochemical processes such as greenhouse gas fluxes across the water-atmosphere boundary. Limnic systems are known to be super-saturated with greenhouse gases [40]. While global CO_2_ emissions from freshwater have been estimated to 0.75 Pg y^−1^ [41] (equal to oceanic emissions [15]), these estimates do not include emissions following ice-off. Sediments of freshwater systems store ca. 3 times more carbon (0.3 Pg y^−1^) than marine ones (0.1 Pg y^−1^) (∼150 times more when normalized to area) [16]. Yet, this figure does not include the unresolved fate of massive under-ice blooms in seasonally ice covered lakes. With the exception of extremely oligotrophic lakes, most of the OM in limnic systems is of autochthonous origin [16]. Therefore, the response of the heterotrophic bacterial community to massive under ice phytoplankton blooms potentially determines whether the lake acts as a C sink or a source. To better understand the role of lakes in global biogeochemical cycles and biodiversity throughout the whole season, it will be crucial to perform year-round studies with a greater focus on the winter season, which - independent of the low temperature - has the potential for high biological activities as long as phototrophic OM remains available for heterotrophic processing. Such studies should have a high temporal sampling resolution (as can be achieved by automatic sampling or monitoring) since as demonstrated in our study the entire bloom event can last less than two weeks. The short duration of such dynamic events points to the fact that monthly routine sampling is very likely to miss such events and that even in winter, boosts in biological activities and OM turnover occur. Extending earlier studies, e.g. those from Lake Baikal [42], usually covered by clear ice, we here suggest that energy and substrate availability rather than temperature are the key factors controlling dynamics of aquatic microorganisms. The dimensions of these carbon driven heterotrophic blooms are important for understanding their present ecological role in aquatic ecosystems, particularly when taking into account that in the future sea ice will rapidly reside [43] and perennially ice-covered aquatic ecosystems may fully disappear [15].

## Supporting Information

Figure S1 Oxygen concentration (A) and PH (B) profiles measured during winter 2010 in Lake Stechlin.
(TIF)

Figure S2 Colonization density of particle-attached bacteria and abundance of free living cells. Since particles vary in size, the cell number (DAPI signal) per particle (a cyanobacteria segment) was normalized according to the particle's area to 100 µm^2^. Median and average values are shown by full and dashed lines, respectively.
(TIFF)

Figure S3 DGGE analysis of the bacterial diversity of the two phases of the bloom separated into particle-attached (PA) and free-living (FL) bacterial communities of epilimnion (EL) and hypolimnion (HL).
(TIF)

Figure S4 Sequence frequencies of major particle-attached and free-living bacterial genera in the epilimnion and hypolimnion of Lake Stechlin during Phase I (A) and Phase II (B) of the under-ice bloom. Genera and clades of not yet cultured bacteria (e.g. Ac1, LD12) are detailed only within families making up over 2% of the total sequences in at least one sample. Numbers in the gray fields refer to all the families that made less than 2% of the total sequences each.
(TIFF)

Table S1 List of samples analyzed for Bacterial Protein Production (BPP), Denaturing Gradient Gel Electrophoresis (DGGE), Tag Pyrosequencing (454) and Fluorescence in situ hybridization (FISH).
(DOCX)

Table S2 Oligonucleotide probes used in this study.
(DOCX)

Table S3 Sequence quality and diversity data for each sample.
(DOCX)

Table S4 List of bacterial tax paths.
(PDF)

Table S5 Under-ice phytoplankton blooms in limnic and marine systems.
(DOC)
