# Supplementary figures and images for: Massive Regime Shifts and High Activity of Heterotrophic Bacteria in an Ice-Covered Lake
Source: PLoS One. 2014 Nov 24;9(11):e113611. doi: 10.1371/journal.pone.0113611 (PMC4242651; doi:10.1371/journal.pone.0113611)

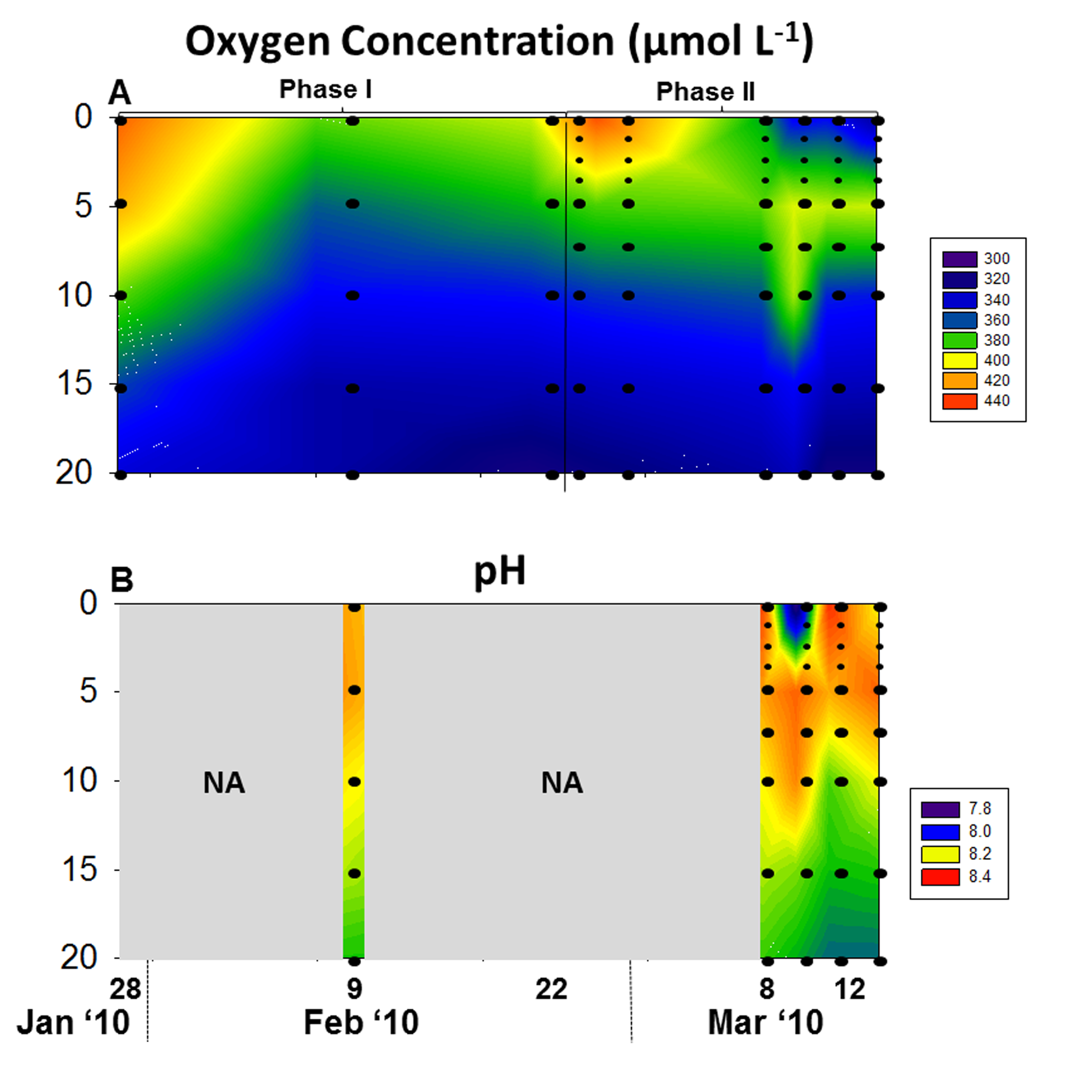

Supplement: Figure S1 — Oxygen concentration (A) and PH (B) profiles measured during winter 2010 in Lake Stechlin. (TIF) [file pone.0113611.s001.tif]

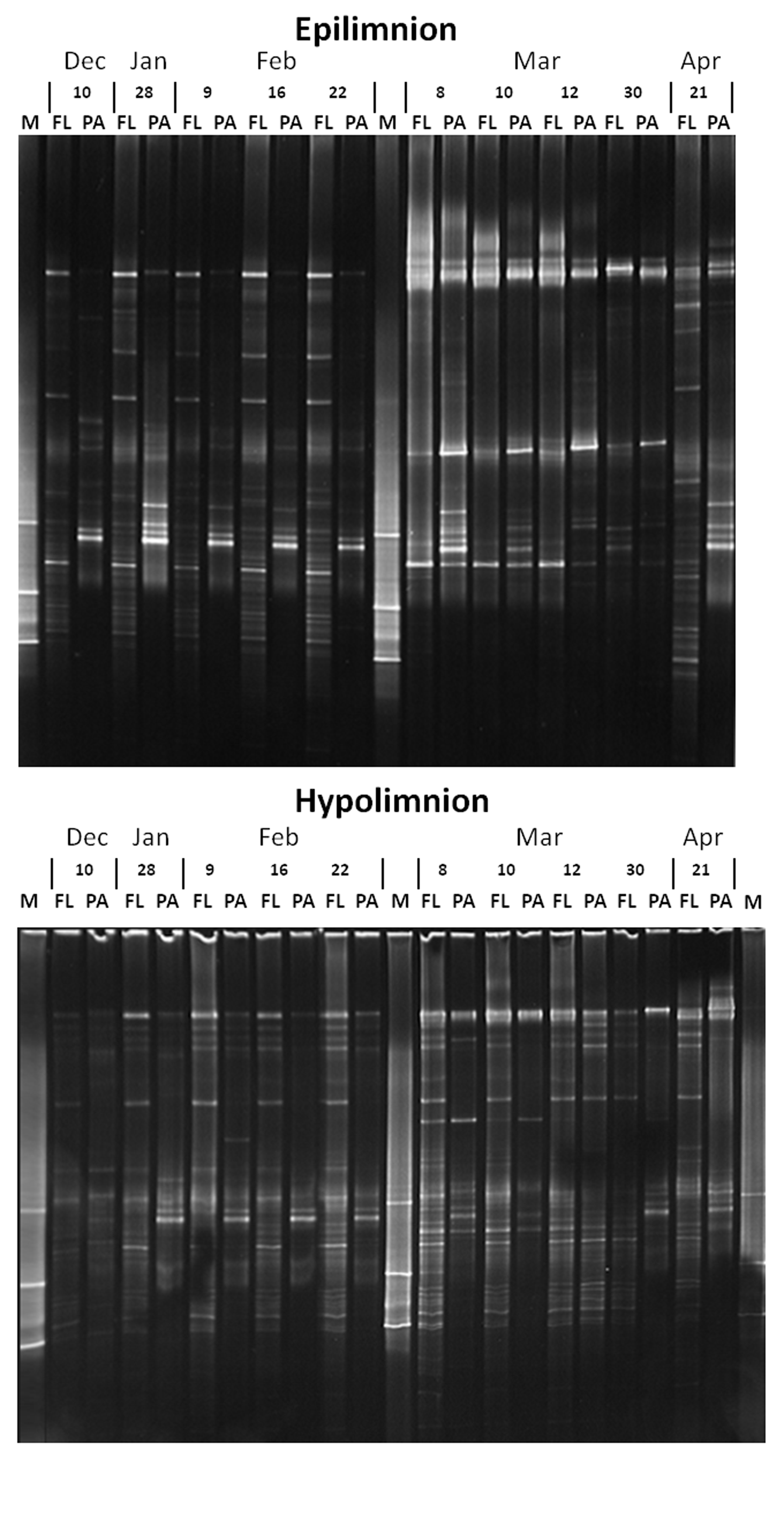

Supplement: Figure S3 — DGGE analysis of the bacterial diversity of the two phases of the bloom separated into particle-attached (PA) and free-living (FL) bacterial communities of epilimnion (EL) and hypolimnion (HL). (TIF) [file pone.0113611.s003.tif]

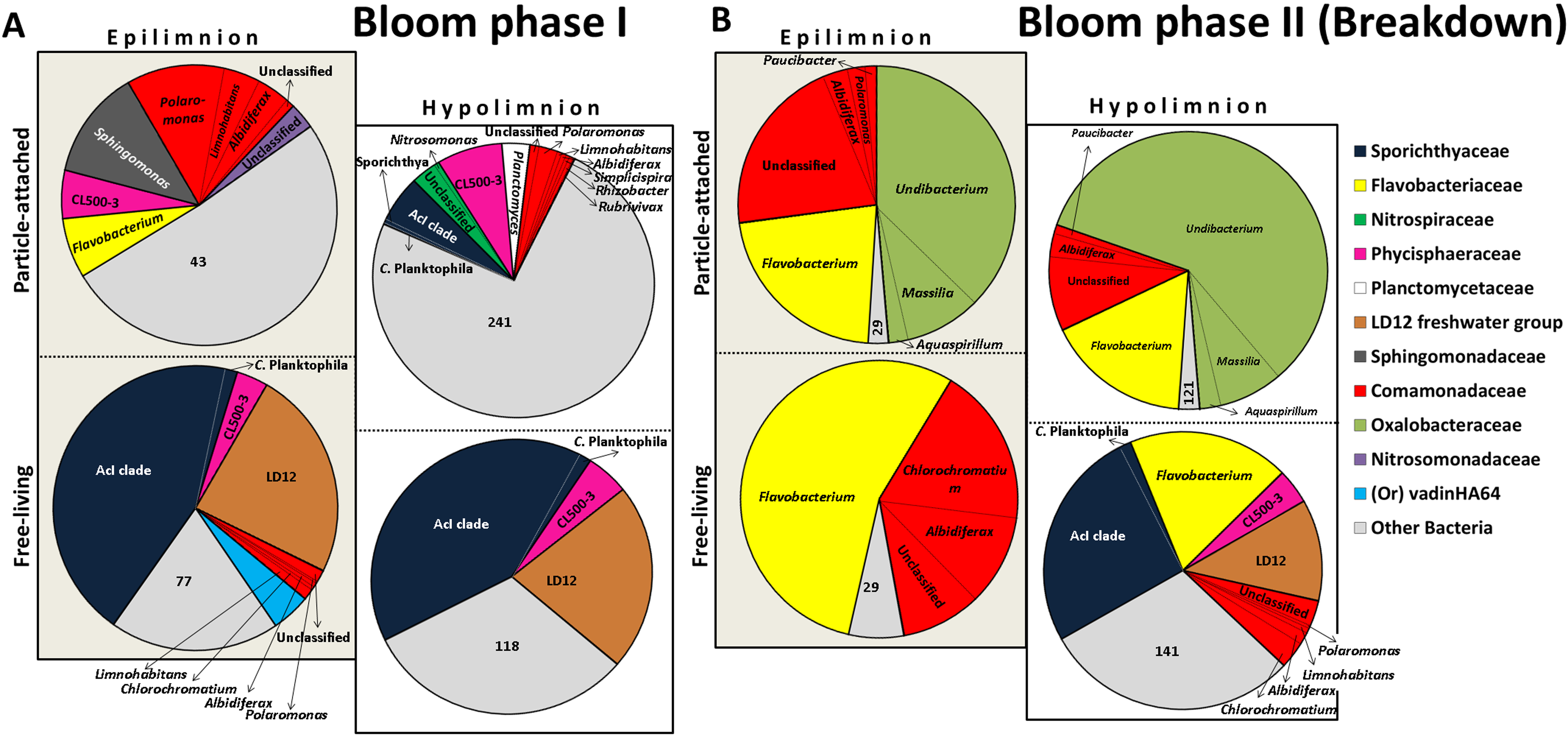

Supplement: Figure S4 — Sequence frequencies of major particle-attached and free-living bacterial genera in the epilimnion and hypolimnion of Lake Stechlin during Phase I (A) and Phase II (B) of the under-ice bloom. Genera and clades of not yet cultured bacteria (e.g. Ac1, LD12) are detailed only within families making up over 2% of the total sequences in at least one sample. Numbers in the gray fields refer to all the families that made less than 2% of the total sequences each. (TIFF) [file pone.0113611.s004.tiff]
