# Supplementary material for: Massive Regime Shifts and High Activity of Heterotrophic Bacteria in an Ice-Covered Lake
Source: PLoS One. 2014 Nov 24;9(11):e113611. doi: 10.1371/journal.pone.0113611 (PMC4242651; doi:10.1371/journal.pone.0113611)
Supplement: Table S1 — List of samples analyzed for Bacterial Protein Production (BPP), Denaturing Gradient Gel Electrophoresis (DGGE), Tag Pyrosequencing (454) and Fluorescence in situ hybridization (FISH). (DOCX) [file pone.0113611.s005.docx]

**Table S1.** List of samples analyzed for Bacterial Protein Production (BPP), Denaturing Gradient Gel Electrophoresis (DGGE), Tag Pyrosequencing (454) and Fluorescence in situ hybridization (FISH)

| **Date** | **BPP** | **DGGE** | **454** | **FISH** | **Stratum** | **Size fraction** |
| --- | --- | --- | --- | --- | --- | --- |
| **10-Dec** | - | + | - | - | **Epilimnion** **(EL)**  & **Hypolimnion (HL)** | **Particle-associated (PA)** > 5 µm &  **Free-living (FL)** 0.2 -5 µm |
| **28-Jan** | + | + | + (Pooled DNA samples) | + |  |  |
| **9-Feb** | + | + |  | + |  |  |
| **16-Feb** | - | + |  | + |  |  |
| **22-Feb** | + | + |  | + |  |  |
| **1-Mar** | + | - | - | - |  |  |
| **8-Mar** | + | + | + (Pooled DNA samples) | + |  |  |
| **10-Mar** | + | + |  | + |  |  |
| **12-Mar** | + | + |  | - |  |  |
| **30-Mar** | - | + |  | - |  |  |
| **21-Apr** | - | + | - | - |  |  |
| **16-Mar** | + | - | - | + |  |  |
