# Supplementary material for: Massive Regime Shifts and High Activity of Heterotrophic Bacteria in an Ice-Covered Lake
Source: PLoS One. 2014 Nov 24;9(11):e113611. doi: 10.1371/journal.pone.0113611 (PMC4242651; doi:10.1371/journal.pone.0113611)
Supplement: Table S2 — Oligonucleotide probes used in this study. (DOCX) [file pone.0113611.s006.docx]

Table S2. Oligonucleotide probes used in this study

| **Probe** | **Target** | **Reference** |
| --- | --- | --- |
| **ALF968** | *Alphaproteobacteria* | [1] |
| **BET42a*** | *Betaproteobacteria* | [2] |
| **GAM42a*** | *Gammaproteobacteria* | [2] |
| **CF319a** | Bacteroidetes | [2] |
| **PLA42a** | Planctomycetes | [3] |
| **HGC69a** | Actinobacteria | [4] |
| **EUB338 I-III^a^** | Bacteria | [5,6] |
| **NON338** | Control | [7] |

^a^ equimolar concentrations of probes EUB338 I, EUB338 II and EUB338 III

* used in combination with a competitor

1. Neef A (1997) Anwendung der in situ-Einzelzell-Identifizierung von Bakterien zur Populationsanalyse in komplexen mikrobiellen Biozönosen Technische Universität München.

2. Manz W, Amann R, Ludwig W, Wagner M, Schleifer K-H (1992) Phylogenetic Oligodeoxynucleotide Probes for the Major Subclasses of Proteobacteria: Problems and Solutions. Syst Appl Microbiol 15: 593–600. doi:10.1016/S0723-2020(11)80121-9.

3. Neef A, Amann R, Schlesner H, Schleifer K (1998) Monitoring a widespread bacterial group: in situ detection of planctomycetes with 16S rRNA-targeted probes. Microbiology 144: 3257–3266. doi:10.1099/00221287-144-12-3257.

4. Roller C, Wagner M, Amann R, Ludwig W, Schleifer K-H (1994) In situ probing of Gram-positive bacteria with high DNA G + C content using 23S rRNA-targeted oligonucleotides. Microbiology 140: 2849–2858. doi:10.1099/00221287-140-10-2849.

5. Amann R, Binder B (1990) Combination of 16S rRNA-targeted oligonucleotide probes with flow cytometry for analyzing mixed microbial populations. Appl Environ Microbiol 56: 1919–1925.

6. Daims H, Brühl A, Amann R (1999) The Domain-specific Probe EUB338 is Insufficient for the Detection of all Bacteria: Development and Evaluation of a more Comprehensive Probe Set. Syst Appl Microbiol 22: 434–444.

7. Wallner G, Amann R, Beisker W (1993) Optimizing fluorescent in situ hybridization with rRNA targeted oligonucleotide probes for flow cytometric identification of microorganisms. Cytometry 14: 136–143.
