# Supplementary material for: Massive Regime Shifts and High Activity of Heterotrophic Bacteria in an Ice-Covered Lake
Source: PLoS One. 2014 Nov 24;9(11):e113611. doi: 10.1371/journal.pone.0113611 (PMC4242651; doi:10.1371/journal.pone.0113611)
Supplement: Table S3 — Sequence quality and diversity data for each sample. (DOCX) [file pone.0113611.s007.docx]

**Table S3.** Sequence quality and diversity data for each sample

| **Sample name** | **# Sequences** | **Minimum length** | **Average length** | **maximum length** | **# OTU^1^** | **Clustered** | **Replicates** | **Rejected** | **Goods coverage** | **Shannon-diversity index (H)^2^** | **Shannon-Evenness (E_H_)** |
| --- | --- | --- | --- | --- | --- | --- | --- | --- | --- | --- | --- |
| **Phase(I)-EL-PA** | 14249 | 150 | 452 | 540 | 300 | 5513 | 8420 | 16 | 0.99 | 3.10 | 0.79 |
| **Phase(I)-EL-FL** | 4195 | 150 | 418 | 531 | 516 | 2312 | 1364 | 3 | 0.95 | 2.06 | 0.47 |
| **Phase(II)-EL-PA** | 9095 | 150 | 420 | 531 | 530 | 4809 | 3735 | 21 | 0.98 | 1.15 | 0.33 |
| **Phase(II)-EL-FL** | 9230 | 150 | 416 | 530 | 520 | 4839 | 3848 | 23 | 0.98 | 0.99 | 0.29 |
| **Phase(I)-HL-PA** | 20728 | 150 | 448 | 563 | 1594 | 8703 | 10382 | 49 | 0.96 | 4.46 | 0.81 |
| **Phase(I)-HL-FL** | 13329 | 150 | 417 | 556 | 1125 | 7169 | 5001 | 34 | 0.97 | 2.30 | 0.48 |
| **Phase(II)-HL-PA** | 42603 | 150 | 417 | 544 | 1343 | 19311 | 21854 | 95 | 0.99 | 0.98 | 0.20 |
| **Phase(II)-HL-FL** | 19506 | 150 | 421 | 546 | 1700 | 11004 | 6753 | 49 | 0.97 | 2.66 | 0.53 |
| ^1^ OTUs were clustered at 98% sequence similarity | | | | | | | | | | |  |
| ^2^ Based on differences in #seq, H was calculated for random subset of 4000 sequences | | | | | | | | | | |  |
