# Supplementary material for: Massive Regime Shifts and High Activity of Heterotrophic Bacteria in an Ice-Covered Lake
Source: PLoS One. 2014 Nov 24;9(11):e113611. doi: 10.1371/journal.pone.0113611 (PMC4242651; doi:10.1371/journal.pone.0113611)
Supplement: Table S4 — List of bacterial tax paths. (PDF) [file pone.0113611.s008.pdf]

Phase(I)-EL-PA	Phase(I)-EL-FL	Phase(II)-EL-PA	Phase(II)-EL-FL	Phase(I)-HL-PA	Phase(I)-HL-FL	Phase(II)-HL-PA	Phase(II)-HL-FL	Domain	Phylum	Class	Order	Family	Genus
0.00	0.00	0.00	0.00	0.05	0.00	0.00	0.01	Bacteria	Acidobacteria	Acidobacteria	45597		
0.00	0.00	0.00	0.00	0.02	0.00	0.00	0.00	Bacteria	Acidobacteria	Acidobacteria	Acidobacteriales	Acidobacteriaceae	Granulicella
0.00	0.00	0.00	0.00	0.12	0.00	0.00	0.00	Bacteria	Acidobacteria	Acidobacteria	BPC102		
2.14	0.00	0.03	0.00	0.35	0.02	0.02	0.01	Bacteria	Acidobacteria	Acidobacteria	Candidatus Chloracidobacterium		
0.00	0.00	0.00	0.00	0.00	0.01	0.02	0.01	Bacteria	Acidobacteria	Acidobacteria	Candidatus Solibacter		
0.00	0.07	0.00	0.00	0.70	0.08	0.01	0.09	Bacteria	Acidobacteria	Acidobacteria	DA023		
0.00	0.00	0.00	0.00	0.00	0.00	0.00	0.02	Bacteria	Acidobacteria	Acidobacteria	DA052		
0.00	0.15	0.00	0.00	0.00	0.14	0.00	0.04	Bacteria	Acidobacteria	Acidobacteria	Order Incertae Sedis	Family Incertae Sedis	Bryobacter
0.00	0.00	0.00	0.00	0.02	0.00	0.00	0.02	Bacteria	Acidobacteria	Acidobacteria	SJA-149		
0.00	0.00	0.00	0.00	0.05	0.00	0.01	0.00	Bacteria	Acidobacteria	Holophagae	32-20		
0.00	0.00	0.00	0.00	0.17	0.00	0.00	0.00	Bacteria	Acidobacteria	Holophagae	FW34		
0.00	0.29	0.00	0.00	0.30	0.31	0.00	0.19	Bacteria	Acidobacteria	Holophagae	Holophagales	Holophagaceae	marine group
0.00	0.00	0.00	0.00	0.42	0.01	0.04	0.06	Bacteria	Acidobacteria	Holophagae	Holophagales	Holophagaceae	uncultured
0.00	0.00	0.00	0.00	0.05	0.00	0.00	0.00	Bacteria	Acidobacteria	Holophagae	NKB17		
0.00	0.00	0.00	0.00	0.00	0.00	0.00	0.00	Bacteria	Acidobacteria	Holophagae	iii1-8		
0.00	0.00	0.00	0.00	0.02	0.00	0.00	0.00	Bacteria	Acidobacteria	RB25			
0.00	0.90	0.00	0.08	0.60	1.27	0.01	1.14	Bacteria	Actinobacteria	Acidimicrobiia	Acidimicrobiales	Acidimicrobiaceae	CL500-29 marine group
0.00	0.00	0.00	0.00	0.05	0.00	0.00	0.00	Bacteria	Actinobacteria	Acidimicrobiia	Acidimicrobiales	Acidimicrobiaceae	uncultured
1.29	0.02	0.00	0.00	0.22	0.01	0.03	0.01	Bacteria	Actinobacteria	Acidimicrobiia	Acidimicrobiales	Candidatus Microthrix	
0.00	0.00	0.00	0.00	0.05	0.00	0.00	0.00	Bacteria	Actinobacteria	Acidimicrobiia	Acidimicrobiales	Iamiaceae	Iamia
0.00	0.00	0.00	0.00	0.05	0.00	0.00	0.00	Bacteria	Actinobacteria	Acidimicrobiia	Acidimicrobiales	OCS155 marine group	
0.00	0.17	0.01	0.01	0.30	0.24	0.01	0.22	Bacteria	Actinobacteria	Acidimicrobiia	Acidimicrobiales	TM214	
0.00	0.00	0.00	0.00	0.20	0.02	0.01	0.02	Bacteria	Actinobacteria	Acidimicrobiia	Acidimicrobiales	uncultured	
0.00	0.00	0.00	0.00	0.15	0.00	0.01	0.04	Bacteria	Actinobacteria	Actinobacteria	Corynebacteriales	Mycobacteriaceae	Mycobacterium
0.00	0.00	0.00	0.00	0.02	0.00	0.00	0.00	Bacteria	Actinobacteria	Actinobacteria	Corynebacteriales	Nocardiaceae	Nocardia
0.00	0.00	0.00	0.00	0.00	0.00	0.00	0.00	Bacteria	Actinobacteria	Actinobacteria	Corynebacteriales	Nocardiaceae	Rhodococcus
0.00	0.00	0.00	0.00	0.02	0.00	0.00	0.00	Bacteria	Actinobacteria	Actinobacteria	Frankiales	Cryptosporangiaceae	Fodinicola
0.00	0.00	0.00	0.00	0.12	0.00	0.00	0.00	Bacteria	Actinobacteria	Actinobacteria	Frankiales	Frankiaceae	Frankia
0.00	0.00	0.00	0.00	0.10	0.00	0.00	0.00	Bacteria	Actinobacteria	Actinobacteria	Frankiales	Geodermatophilaceae	Blastococcus
0.00	1.38	0.00	0.03	0.15	1.27	0.01	0.93	Bacteria	Actinobacteria	Actinobacteria	Frankiales	Sporichthyaceae	Candidatus Planktophila
0.00	0.00	0.00	0.00	0.25	0.05	0.00	0.03	Bacteria	Actinobacteria	Actinobacteria	Frankiales	Sporichthyaceae	Sporichthya
1.72	43.62	0.00	1.00	5.80	40.29	0.02	26.05	Bacteria	Actinobacteria	Actinobacteria	Frankiales	Sporichthyaceae	hgcI clade
0.00	0.00	0.00	0.00	0.02	0.00	0.00	0.00	Bacteria	Actinobacteria	Actinobacteria	Frankiales	Sporichthyaceae	uncultured
0.00	0.00	0.00	0.00	0.07	0.00	0.00	0.00	Bacteria	Actinobacteria	Actinobacteria	Micrococcales	Dermatophilaceae	uncultured
0.00	0.00	0.00	0.00	0.07	0.00	0.00	0.00	Bacteria	Actinobacteria	Actinobacteria	Micrococcales	Intrasporangiaceae	Humibacillus
0.00	0.00	0.00	0.00	0.00	0.00	0.00	0.01	Bacteria	Actinobacteria	Actinobacteria	Micrococcales	Intrasporangiaceae	Janibacter
0.00	0.00	0.00	0.00	0.00	0.00	0.00	0.00	Bacteria	Actinobacteria	Actinobacteria	Micrococcales	Microbacteriaceae	Agrococcus
0.00	0.00	0.00	0.00	0.00	0.01	0.00	0.00	Bacteria	Actinobacteria	Actinobacteria	Micrococcales	Microbacteriaceae	Alpinimonas
0.00	0.02	0.00	0.00	0.00	0.00	0.00	0.00	Bacteria	Actinobacteria	Actinobacteria	Micrococcales	Microbacteriaceae	Candidatus Aquiluna
0.00	0.00	0.00	0.00	0.00	0.00	0.00	0.03	Bacteria	Actinobacteria	Actinobacteria	Micrococcales	Microbacteriaceae	Candidatus Limnoluna
0.00	0.02	0.00	0.00	0.02	0.00	0.00	0.00	Bacteria	Actinobacteria	Actinobacteria	Micrococcales	Microbacteriaceae	Candidatus Rhodoluna
0.00	0.00	0.00	0.00	0.00	0.00	0.01	0.00	Bacteria	Actinobacteria	Actinobacteria	Micrococcales	Microbacteriaceae	Leifsonia
0.00	0.00	0.00	0.00	0.00	0.00	0.01	0.01	Bacteria	Actinobacteria	Actinobacteria	Micrococcales	Microbacteriaceae	S24526
0.00	0.00	0.00	0.00	0.00	0.00	0.00	0.01	Bacteria	Actinobacteria	Actinobacteria	Micrococcales	Microbacteriaceae	Yonghaparkia
0.00	0.00	0.00	0.00	0.00	0.01	0.00	0.03	Bacteria	Actinobacteria	Actinobacteria	Micrococcales	Micrococcaceae	Arthrobacter
0.86	0.22	0.00	0.00	0.12	0.56	0.00	0.59	Bacteria	Actinobacteria	Actinobacteria	PeM15		
0.00	0.00	0.00	0.00	0.05	0.00	0.00	0.00	Bacteria	Actinobacteria	Actinobacteria	Propionibacteriales	Nocardioidaceae	Nocardioides
0.00	0.00	0.00	0.00	0.00	0.05	0.01	0.00	Bacteria	Actinobacteria	Actinobacteria	Propionibacteriales	Propionibacteriaceae	Propionibacterium
0.00	0.00	0.01	0.00	0.00	0.00	0.00	0.00	Bacteria	Actinobacteria	Actinobacteria	Pseudonocardiales	Pseudonocardiaceae	Pseudonocardia
0.00	0.00	0.00	0.00	0.00	0.01	0.00	0.00	Bacteria	Actinobacteria	Actinobacteria	Streptomycetales	Streptomycetaceae	Streptomyces
0.00	0.00	0.00	0.00	0.00	0.01	0.00	0.00	Bacteria	Actinobacteria	Actinobacteria	Streptosporangiales	Streptosporangiaceae	Streptosporangium
0.00	0.00	0.00	0.00	0.12	0.00	0.00	0.00	Bacteria	Actinobacteria	MB-A2-108			

Phase(I)-EL-PA	Phase(I)-EL-FL	Phase(II)-EL-PA	Phase(II)-EL-FL	Phase(I)-HL-PA	Phase(I)-HL-FL	Phase(II)-HL-PA	Phase(II)-HL-FL	Domain	Phylum	Class	Order	Family	Genus
0.00	0.00	0.00	0.00	0.32	0.00	0.00	0.00	Bacteria	Actinobacteria	OPB41			
0.00	0.00	0.00	0.00	0.10	0.00	0.00	0.01	Bacteria	Actinobacteria	Thermoleophilia	Gaiellales	Gaiellaceae	Gaiella
0.86	0.32	0.00	0.00	0.22	0.41	0.00	0.42	Bacteria	Actinobacteria	Thermoleophilia	Gaiellales	uncultured	
0.00	0.05	0.00	0.00	0.07	0.10	0.00	0.26	Bacteria	Actinobacteria	Thermoleophilia	Solirubrobacterales	0319-6M6	
0.00	0.02	0.00	0.00	0.20	0.08	0.01	0.07	Bacteria	Actinobacteria	Thermoleophilia	Solirubrobacterales	480-2	
0.00	0.00	0.00	0.00	0.00	0.00	0.00	0.01	Bacteria	Actinobacteria	Thermoleophilia	Solirubrobacterales	Patulibacteraceae	Patulibacter
0.00	0.00	0.00	0.00	0.00	0.01	0.00	0.00	Bacteria	Actinobacteria	Thermoleophilia	Solirubrobacterales	TM146	
0.00	0.00	0.00	0.00	0.20	0.00	0.00	0.00	Bacteria	Armatimonadetes				
0.43	0.00	0.00	0.00	0.05	0.06	0.00	0.05	Bacteria	Armatimonadetes	Armatimonadia	Armatimonadales	Armatimonadaceae	Armatimonas
0.00	0.00	0.00	0.00	0.05	0.00	0.00	0.00	Bacteria	Armatimonadetes	Chthonomonadetes	Chthonomonadales	Chthonomonadaceae	Chthonomonas
0.00	0.00	0.00	0.00	0.10	0.02	0.00	0.01	Bacteria	BD1-5				
0.00	0.00	0.00	0.00	0.05	0.00	0.00	0.00	Bacteria	BHI80-139				
0.00	0.00	0.00	0.00	0.05	0.00	0.00	0.00	Bacteria	Bacteroidetes	BD2-2			
0.00	0.00	0.00	0.00	0.02	0.00	0.00	0.00	Bacteria	Bacteroidetes	BSV13			
0.00	0.00	0.00	0.00	0.02	0.00	0.00	0.00	Bacteria	Bacteroidetes	Bacteroidia	Bacteroidales	Rikenellaceae	Blvii28 wastewater-sludge group
0.00	0.05	0.00	0.00	0.05	0.09	0.00	0.07	Bacteria	Bacteroidetes	Cytophagia	Cytophagales	Cyclobacteriaceae	uncultured
0.00	0.00	0.00	0.00	0.00	0.00	0.00	0.00	Bacteria	Bacteroidetes	Cytophagia	Cytophagales	Cytophagaceae	Adhaeribacter
0.00	0.34	0.00	0.01	0.05	0.19	0.00	0.16	Bacteria	Bacteroidetes	Cytophagia	Cytophagales	Cytophagaceae	Arcicella
0.43	0.00	0.00	0.00	0.37	0.00	0.00	0.00	Bacteria	Bacteroidetes	Cytophagia	Cytophagales	Cytophagaceae	Flexibacter
0.00	0.00	0.00	0.00	0.02	0.00	0.00	0.01	Bacteria	Bacteroidetes	Cytophagia	Cytophagales	Cytophagaceae	Hymenobacter
0.00	0.00	0.00	0.00	0.02	0.00	0.00	0.00	Bacteria	Bacteroidetes	Cytophagia	Cytophagales	Cytophagaceae	Leadbetterella
0.00	0.00	0.00	0.00	0.02	0.00	0.00	0.00	Bacteria	Bacteroidetes	Cytophagia	Cytophagales	Cytophagaceae	Runella
0.00	0.00	0.00	0.00	0.05	0.00	0.00	0.00	Bacteria	Bacteroidetes	Cytophagia	Cytophagales	Cytophagaceae	Spirosoma
0.43	0.00	0.00	0.00	0.00	0.00	0.00	0.00	Bacteria	Bacteroidetes	Cytophagia	Cytophagales	Flammeovirgaceae	Candidatus Amoebophilus
0.86	0.00	0.00	0.00	0.00	0.00	0.00	0.00	Bacteria	Bacteroidetes	Cytophagia	Cytophagales	Flammeovirgaceae	Candidatus Cardinium
0.00	0.00	0.00	0.00	0.02	0.00	0.00	0.00	Bacteria	Bacteroidetes	Cytophagia	Order III Incertae Sedis	MAT-CR-P4-C12	
0.86	1.26	0.00	0.03	1.32	0.74	0.00	0.37	Bacteria	Bacteroidetes	Flavobacteria	Flavobacteriales	Cryomorphaceae	Fluviicola
0.00	0.00	0.00	0.00	0.02	0.00	0.00	0.00	Bacteria	Bacteroidetes	Flavobacteria	Flavobacteriales	Flavobacteriaceae	Chryseobacterium
6.87	0.27	21.89	55.12	2.07	0.10	16.82	18.78	Bacteria	Bacteroidetes	Flavobacteria	Flavobacteriales	Flavobacteriaceae	Flavobacterium
0.00	0.00	0.00	0.00	0.00	0.02	0.00	0.01	Bacteria	Bacteroidetes	Flavobacteria	Flavobacteriales	Flavobacteriaceae	NS3a marine group
0.00	0.00	0.00	0.00	0.00	0.00	0.00	0.00	Bacteria	Bacteroidetes	Flavobacteria	Flavobacteriales	Flavobacteriaceae	NS5 marine group
0.00	0.00	0.00	0.00	0.00	0.00	0.00	0.01	Bacteria	Bacteroidetes	Flavobacteria	Flavobacteriales	Flavobacteriaceae	uncultured
0.00	0.02	0.00	0.00	0.20	0.13	0.01	0.02	Bacteria	Bacteroidetes	Flavobacteria	Flavobacteriales	NS9 marine group	
0.00	0.00	0.00	0.00	0.00	0.00	0.00	0.00	Bacteria	Bacteroidetes	Flavobacteria	Flavobacteriales	R103-B20	
0.00	0.00	0.00	0.00	0.07	0.00	0.00	0.00	Bacteria	Bacteroidetes	SB-1			
0.00	0.00	0.03	0.00	0.05	0.00	0.00	0.00	Bacteria	Bacteroidetes	SB-5			
0.00	0.00	0.00	0.00	0.05	0.00	0.00	0.00	Bacteria	Bacteroidetes	Sphingobacteriia	Sphingobacteriales		
0.00	0.00	0.00	0.00	0.00	0.00	0.00	0.00	Bacteria	Bacteroidetes	Sphingobacteriia	Sphingobacteriales	AKYH767	
0.86	0.87	0.00	0.00	0.47	0.53	0.02	0.38	Bacteria	Bacteroidetes	Sphingobacteriia	Sphingobacteriales	Chitinophagaceae	Ferruginibacter
0.43	0.00	0.00	0.00	0.10	0.00	0.00	0.00	Bacteria	Bacteroidetes	Sphingobacteriia	Sphingobacteriales	Chitinophagaceae	Filimonas
0.00	0.19	0.00	0.00	0.15	0.02	0.00	0.08	Bacteria	Bacteroidetes	Sphingobacteriia	Sphingobacteriales	Chitinophagaceae	Hydrotalea
0.86	0.83	0.00	0.01	0.37	0.40	0.00	0.46	Bacteria	Bacteroidetes	Sphingobacteriia	Sphingobacteriales	Chitinophagaceae	Sediminibacterium
0.00	0.00	0.00	0.00	0.00	0.00	0.00	0.00	Bacteria	Bacteroidetes	Sphingobacteriia	Sphingobacteriales	Chitinophagaceae	Segetibacter
0.00	0.24	0.00	0.00	0.25	0.16	0.01	0.14	Bacteria	Bacteroidetes	Sphingobacteriia	Sphingobacteriales	Chitinophagaceae	uncultured
0.00	0.00	0.00	0.00	0.00	0.00	0.00	0.01	Bacteria	Bacteroidetes	Sphingobacteriia	Sphingobacteriales	FFCH9454	
0.00	0.00	0.00	0.00	0.02	0.00	0.00	0.00	Bacteria	Bacteroidetes	Sphingobacteriia	Sphingobacteriales	KD1-131	
0.00	0.39	0.01	0.00	0.37	0.49	0.03	0.25	Bacteria	Bacteroidetes	Sphingobacteriia	Sphingobacteriales	KD3-93	
1.29	0.02	0.00	0.01	0.15	0.04	0.00	0.04	Bacteria	Bacteroidetes	Sphingobacteriia	Sphingobacteriales	LiUU-11-161	
0.00	0.15	0.00	0.00	0.02	0.07	0.00	0.08	Bacteria	Bacteroidetes	Sphingobacteriia	Sphingobacteriales	NS11-12 marine group	
2.14	0.05	0.00	0.00	0.72	0.03	0.01	0.01	Bacteria	Bacteroidetes	Sphingobacteriia	Sphingobacteriales	PHOS-HE51	
0.00	0.00	0.00	0.00	0.02	0.00	0.00	0.00	Bacteria	Bacteroidetes	Sphingobacteriia	Sphingobacteriales	ST-12K33	
0.00	0.00	0.01	0.00	0.12	0.02	0.03	0.00	Bacteria	Bacteroidetes	Sphingobacteriia	Sphingobacteriales	Saprospiraceae	Candidatus Aquirestis

Phase(I)-EL-PA	Phase(I)-EL-FL	Phase(II)-EL-PA	Phase(II)-EL-FL	Phase(I)-HL-PA	Phase(I)-HL-FL	Phase(II)-HL-PA	Phase(II)-HL-FL	Domain	Phylum	Class	Order	Family	Genus
0.00	0.00	0.00	0.00	0.12	0.00	0.00	0.00	Bacteria	Bacteroidetes	Sphingobacteriia	Sphingobacteriales	Saprospiraceae	uncultured
0.00	0.85	0.00	0.00	0.02	0.64	0.00	0.59	Bacteria	Bacteroidetes	Sphingobacteriia	Sphingobacteriales	Sphingobacteriaceae	Pedobacter
0.00	0.07	0.00	0.00	0.00	0.01	0.00	0.03	Bacteria	Bacteroidetes	Sphingobacteriia	Sphingobacteriales	Sphingobacteriaceae	Solitalea
0.00	0.00	0.00	0.00	0.17	0.00	0.00	0.00	Bacteria	Bacteroidetes	Sphingobacteriia	Sphingobacteriales	WCHB1-69	
1.72	0.34	0.00	0.01	1.44	0.68	0.01	0.57	Bacteria	Bacteroidetes	Sphingobacteriia	Sphingobacteriales	env.OPS 17	
0.00	0.00	0.00	0.00	0.05	0.00	0.00	0.00	Bacteria	Bacteroidetes	VC2.1 Bac22			
0.00	0.00	0.00	0.00	0.72	0.00	0.00	0.00	Bacteria	Bacteroidetes	vadinHA17			
0.00	0.00	0.00	0.00	0.27	0.00	0.01	0.00	Bacteria	Candidate division BRC1				
0.00	0.00	0.00	0.00	0.05	0.00	0.00	0.01	Bacteria	Candidate division OD1				
0.00	0.00	0.00	0.00	0.02	0.00	0.01	0.00	Bacteria	Candidate division OP11				
0.00	0.00	0.00	0.00	0.22	0.02	0.00	0.00	Bacteria	Candidate division OP3				
0.00	0.00	0.00	0.00	0.02	0.00	0.00	0.00	Bacteria	Candidate division OP9				
0.00	0.00	0.00	0.00	0.05	0.00	0.00	0.00	Bacteria	Candidate division SR1				
0.00	0.02	0.00	0.01	0.22	0.06	0.00	0.07	Bacteria	Candidate division TM7				
0.00	0.00	0.00	0.00	0.42	0.00	0.00	0.00	Bacteria	Candidate division WS3				
0.43	0.56	0.03	0.02	0.75	0.43	0.01	0.51	Bacteria	Chlorobi	Chlorobia	Chlorobiales	OPB56	
0.00	0.00	0.00	0.00	0.05	0.00	0.00	0.00	Bacteria	Chlorobi	Chlorobia	Chlorobiales	SJA-28	
0.00	0.00	0.00	0.00	0.55	0.00	0.00	0.00	Bacteria	Chlorobi	Ignavibacteria	Ignavibacteriales	BSV26	
0.00	0.00	0.00	0.00	0.10	0.00	0.00	0.00	Bacteria	Chlorobi	Ignavibacteria	Ignavibacteriales	Ignavibacteriaceae	Ignavibacterium
0.00	0.00	0.00	0.00	0.10	0.00	0.00	0.00	Bacteria	Chlorobi	Ignavibacteria	Ignavibacteriales	LD-RB-34	
0.00	0.00	0.00	0.00	0.10	0.00	0.00	0.00	Bacteria	Chlorobi	Ignavibacteria	Ignavibacteriales	PHOS-HE36	
0.00	0.00	0.00	0.00	0.02	0.00	0.00	0.00	Bacteria	Chlorobi	Ignavibacteria	Ignavibacteriales	SR-FBR-L83	
0.00	0.00	0.00	0.00	0.22	0.00	0.00	0.00	Bacteria	Chloroflexi	Anaerolineae	Anaerolineales	Anaerolineaceae	Anaerolinea
0.43	0.70	0.00	0.00	2.09	2.04	0.01	2.21	Bacteria	Chloroflexi	Anaerolineae	Anaerolineales	Anaerolineaceae	uncultured
0.00	0.00	0.00	0.00	0.07	0.01	0.00	0.00	Bacteria	Chloroflexi	Caldilineae	Caldilineales	Caldilineaceae	Caldilinea
0.00	0.00	0.00	0.00	0.47	0.00	0.01	0.01	Bacteria	Chloroflexi	Caldilineae	Caldilineales	Caldilineaceae	uncultured
0.43	0.12	0.01	0.00	0.05	0.11	0.00	0.06	Bacteria	Chloroflexi	Chloroflexi	Chloroflexales	Chloroflexaceae	Roseiflexus
0.00	0.00	0.00	0.00	0.05	0.00	0.00	0.00	Bacteria	Chloroflexi	Dehalococcoidetes	Order Incertae Sedis	Family Incertae Sedis	Dehalococcoides
0.00	0.00	0.00	0.00	0.02	0.00	0.00	0.00	Bacteria	Chloroflexi	FS117-23B-02			
0.00	0.00	0.00	0.00	0.02	0.00	0.00	0.00	Bacteria	Chloroflexi	GIF3			
0.00	0.00	0.00	0.00	0.97	0.00	0.00	0.00	Bacteria	Chloroflexi	GIF9			
0.00	0.05	0.00	0.00	0.07	0.01	0.00	0.04	Bacteria	Chloroflexi	JG30-KF-CM66			
0.00	0.00	0.00	0.00	0.25	0.00	0.00	0.02	Bacteria	Chloroflexi	KD4-96			
0.00	0.00	0.00	0.00	0.02	0.00	0.00	0.00	Bacteria	Chloroflexi	Ktedonobacteria	C0119		
0.00	0.00	0.00	0.00	0.07	0.00	0.00	0.00	Bacteria	Chloroflexi	MSB-5B2			
0.00	0.00	0.00	0.00	0.70	0.00	0.00	0.00	Bacteria	Chloroflexi	MSBL5			
0.00	0.00	0.00	0.00	0.02	0.00	0.00	0.00	Bacteria	Chloroflexi	Napoli-4B-65			
0.00	0.00	0.00	0.00	0.10	0.00	0.00	0.00	Bacteria	Chloroflexi	S085			
0.00	0.00	0.00	0.00	0.12	0.00	0.00	0.00	Bacteria	Chloroflexi	SHA-26			
0.86	1.53	0.00	0.07	0.55	2.81	0.01	1.81	Bacteria	Chloroflexi	SL56 marine group			
0.43	0.05	0.00	0.00	0.10	0.10	0.01	0.07	Bacteria	Chloroflexi	TK10			
0.00	0.00	0.00	0.00	0.10	0.02	0.00	0.01	Bacteria	Chloroflexi	Thermomicrobia	JG30-KF-CM45		
0.00	0.00	0.00	0.00	0.20	0.00	0.00	0.00	Bacteria	Chloroflexi	vadinBA26			
0.00	0.00	0.00	0.00	0.02	0.00	0.00	0.00	Bacteria	Deferribacteres	Deferribacteres	Deferribacterales	Family Incertae Sedis	Caldithrix
0.00	0.00	0.00	0.00	0.02	0.00	0.00	0.00	Bacteria	Deferribacteres	Deferribacteres	Deferribacterales	LCP-89	
0.00	0.00	0.00	0.00	0.00	0.00	0.01	0.00	Bacteria	Deinococcus-Thermus	Deinococci	Deinococcales	Deinococcaceae	Deinococcus
0.00	0.00	0.00	0.00	0.02	0.00	0.00	0.00	Bacteria	Deinococcus-Thermus	Deinococci	Deinococcales	Trueperaceae	Truepera
0.00	0.00	0.00	0.00	0.17	0.00	0.00	0.00	Bacteria	Deinococcus-Thermus	Deinococci	KD3-62		
0.00	0.00	0.00	0.00	0.02	0.00	0.00	0.00	Bacteria	Elusimicrobia	Elusimicrobia	Lineage IIa		
0.00	0.00	0.00	0.00	0.02	0.00	0.00	0.00	Bacteria	Elusimicrobia	Elusimicrobia	Lineage IIb		
0.00	0.00	0.00	0.00	0.07	0.00	0.00	0.00	Bacteria	Elusimicrobia	Elusimicrobia	Lineage IIc		
0.00	0.00	0.00	0.00	0.05	0.00	0.00	0.00	Bacteria	Fibrobacteres	Fibrobacteria	Fibrobacterales	B122	

Phase(I)-EL-PA	Phase(I)-EL-FL	Phase(II)-EL-PA	Phase(II)-EL-FL	Phase(I)-HL-PA	Phase(I)-HL-FL	Phase(II)-HL-PA	Phase(II)-HL-FL	Domain	Phylum	Class	Order	Family	Genus
0.00	0.00	0.00	0.00	0.07	0.00	0.00	0.00	Bacteria	Firmicutes	Bacilli	Bacillales	Bacillaceae	Bacillus
0.00	0.00	0.00	0.00	0.00	0.01	0.00	0.01	Bacteria	Firmicutes	Bacilli	Bacillales	Paenibacillaceae	Paenibacillus
0.00	0.00	0.00	0.00	0.00	0.00	0.00	0.03	Bacteria	Firmicutes	Bacilli	Bacillales	Staphylococcaceae	Staphylococcus
0.00	0.00	0.00	0.00	0.02	0.00	0.00	0.00	Bacteria	Firmicutes	Bacilli	Lactobacillales	Lactobacillaceae	Lactobacillus
0.00	0.00	0.03	0.00	0.00	0.00	0.00	0.00	Bacteria	Firmicutes	Bacilli	Lactobacillales	Leuconostocaceae	Leuconostoc
0.00	0.00	0.00	0.00	0.00	0.02	0.00	0.00	Bacteria	Firmicutes	Bacilli	Lactobacillales	Streptococcaceae	Streptococcus
0.00	0.07	0.01	0.00	0.00	0.00	0.00	0.00	Bacteria	Firmicutes	Clostridia	Clostridiales	Christensenellaceae	uncultured
0.00	0.00	0.00	0.00	0.02	0.00	0.00	0.00	Bacteria	Firmicutes	Clostridia	Clostridiales	Family XII Incertae Sedis	Acidaminobacter
0.00	0.00	0.00	0.00	0.02	0.00	0.00	0.00	Bacteria	Firmicutes	Clostridia	Clostridiales	Family XIII Incertae Sedis	uncultured
0.00	0.00	0.00	0.00	0.02	0.01	0.00	0.00	Bacteria	Firmicutes	Clostridia	Clostridiales	Family XVIII Incertae Sedis	Symbiobacterium
0.00	0.00	0.03	0.00	0.00	0.00	0.00	0.00	Bacteria	Firmicutes	Clostridia	Clostridiales	Lachnospiraceae	Butyrivibrio
0.00	0.02	0.00	0.00	0.00	0.00	0.00	0.00	Bacteria	Firmicutes	Clostridia	Clostridiales	Lachnospiraceae	Incertae Sedis
0.00	0.00	0.00	0.00	0.02	0.00	0.00	0.00	Bacteria	Firmicutes	Clostridia	Clostridiales	Lachnospiraceae	Roseburia
0.00	0.02	0.00	0.00	0.00	0.01	0.00	0.00	Bacteria	Firmicutes	Clostridia	Clostridiales	Lachnospiraceae	Syntrophococcus
0.00	0.02	0.04	0.00	0.02	0.00	0.00	0.01	Bacteria	Firmicutes	Clostridia	Clostridiales	Lachnospiraceae	uncultured
0.00	0.00	0.00	0.00	0.00	0.00	0.00	0.00	Bacteria	Firmicutes	Clostridia	Clostridiales	Peptostreptococcaceae	Incertae Sedis
0.00	0.00	0.00	0.00	0.02	0.00	0.00	0.00	Bacteria	Firmicutes	Clostridia	Clostridiales	Peptostreptococcaceae	uncultured
0.00	0.00	0.00	0.00	0.10	0.00	0.00	0.00	Bacteria	Firmicutes	Clostridia	Clostridiales	Ruminococcaceae	Incertae Sedis
0.00	0.02	0.03	0.00	0.02	0.00	0.00	0.00	Bacteria	Firmicutes	Clostridia	Clostridiales	Ruminococcaceae	Ruminococcus
0.00	0.02	0.01	0.00	0.00	0.00	0.00	0.00	Bacteria	Firmicutes	Clostridia	Clostridiales	Ruminococcaceae	Saccharofermentans
0.00	0.05	0.09	0.00	0.12	0.02	0.00	0.00	Bacteria	Firmicutes	Clostridia	Clostridiales	Ruminococcaceae	uncultured
0.00	0.00	0.00	0.00	0.00	0.00	0.00	0.00	Bacteria	Firmicutes	Clostridia	Clostridiales	Veillonellaceae	Pelosinus
0.00	0.00	0.00	0.00	0.02	0.00	0.00	0.00	Bacteria	Firmicutes	Clostridia	D8A-2		
0.00	0.00	0.00	0.00	0.02	0.00	0.00	0.00	Bacteria	Firmicutes	Clostridia	Thermoanaerobacterales	Thermoanaerobacteraceae	Gelria
0.00	0.00	0.00	0.00	0.07	0.00	0.00	0.00	Bacteria	GOUTA4				
0.00	0.05	0.00	0.00	0.00	0.03	0.00	0.07	Bacteria	Gemmatimonadetes	Gemmatimonadetes	Gemmatimonadales	Gemmatimonadaceae	
0.00	0.02	0.00	0.00	0.10	0.08	0.01	0.04	Bacteria	Gemmatimonadetes	Gemmatimonadetes	Gemmatimonadales	Gemmatimonadaceae	Gemmatimonas
0.00	0.00	0.00	0.00	0.35	0.00	0.00	0.04	Bacteria	Gemmatimonadetes	Gemmatimonadetes	Gemmatimonadales	Gemmatimonadaceae	uncultured
0.00	0.00	0.00	0.00	0.07	0.00	0.00	0.00	Bacteria	JL-ETNP-Z39				
0.00	0.00	0.00	0.00	0.05	0.00	0.00	0.00	Bacteria	Lentisphaerae	Lentisphaeria	BS5		
0.00	0.00	0.00	0.00	0.10	0.00	0.00	0.00	Bacteria	Lentisphaerae	Lentisphaeria	DEV055		
0.00	0.00	0.00	0.00	0.02	0.00	0.00	0.00	Bacteria	Lentisphaerae	Lentisphaeria	Victivallales	Victivallaceae	Victivallis
0.00	0.00	0.00	0.00	0.02	0.00	0.00	0.00	Bacteria	Lentisphaerae	Lentisphaeria	Victivallales	Victivallaceae	uncultured
0.00	0.00	0.00	0.00	0.00	0.02	0.00	0.00	Bacteria	MVP-21				
0.00	0.00	0.00	0.00	0.07	0.02	0.00	0.01	Bacteria	NPL-UPA2				
0.00	0.00	0.00	0.00	0.10	0.00	0.00	0.00	Bacteria	Nitrospirae	Nitrospira	Nitrospirales	0319-6A21	
0.00	0.00	0.00	0.00	0.07	0.00	0.00	0.00	Bacteria	Nitrospirae	Nitrospira	Nitrospirales	47209	
0.00	0.00	0.00	0.00	2.14	0.00	0.00	0.03	Bacteria	Nitrospirae	Nitrospira	Nitrospirales	Nitrospiraceae	Nitrospira
0.43	0.00	0.00	0.00	1.42	0.00	0.00	0.00	Bacteria	Nitrospirae	Nitrospira	Nitrospirales	Nitrospiraceae	uncultured
0.00	0.00	0.00	0.00	1.10	0.00	0.01	0.00	Bacteria	Nitrospirae	Nitrospira	Nitrospirales	OPB95	
0.00	0.00	0.00	0.00	0.07	0.00	0.00	0.00	Bacteria	OC31				
0.00	0.00	0.00	0.00	0.00	0.00	0.00	0.01	Bacteria	Planctomycetes	BD7-11			
0.00	0.00	0.00	0.00	0.05	0.00	0.00	0.00	Bacteria	Planctomycetes	MBMPE71			
0.00	0.00	0.00	0.00	0.07	0.00	0.00	0.00	Bacteria	Planctomycetes	OM190			
0.00	0.00	0.00	0.00	0.15	0.00	0.00	0.00	Bacteria	Planctomycetes	Phycisphaerae	CCM11a		
0.00	0.00	0.00	0.00	0.12	0.00	0.00	0.00	Bacteria	Planctomycetes	Phycisphaerae	MSBL9		
0.00	0.00	0.00	0.00	0.07	0.00	0.00	0.00	Bacteria	Planctomycetes	Phycisphaerae	ODP1230B30.09		
0.00	0.00	0.00	0.00	0.00	0.00	0.01	0.00	Bacteria	Planctomycetes	Phycisphaerae	Phycisphaerales	08D2Z94 hypersaline microbial mat group	
0.00	0.00	0.00	0.00	0.02	0.00	0.00	0.00	Bacteria	Planctomycetes	Phycisphaerae	Phycisphaerales	AKAU3564 sediment group	
0.00	0.00	0.00	0.00	0.02	0.00	0.00	0.00	Bacteria	Planctomycetes	Phycisphaerae	Phycisphaerales	Phycisphaeraceae	AKYG587
5.58	3.59	0.04	0.05	7.50	5.08	0.15	4.07	Bacteria	Planctomycetes	Phycisphaerae	Phycisphaerales	Phycisphaeraceae	CL500-3
0.00	0.00	0.00	0.00	0.07	0.00	0.00	0.00	Bacteria	Planctomycetes	Phycisphaerae	Phycisphaerales	Phycisphaeraceae	I-8

Phase(I)-EL-PA	Phase(I)-EL-FL	Phase(II)-EL-PA	Phase(II)-EL-FL	Phase(I)-HL-PA	Phase(I)-HL-FL	Phase(II)-HL-PA	Phase(II)-HL-FL	Domain	Phylum	Class	Order	Family	Genus
0.00	0.00	0.00	0.00	0.00	0.00	0.00	0.00	Bacteria	Planctomycetes	Phycisphaerae	Phycisphaerales	Phycisphaeraceae	SM1A02
0.00	0.00	0.00	0.00	0.02	0.00	0.00	0.00	Bacteria	Planctomycetes	Phycisphaerae	Pla1 lineage		
0.00	0.00	0.00	0.00	0.02	0.00	0.00	0.00	Bacteria	Planctomycetes	Phycisphaerae	S-70		
0.00	0.00	0.00	0.00	0.02	0.00	0.00	0.00	Bacteria	Planctomycetes	Phycisphaerae	mle1-8		
0.00	0.00	0.00	0.00	0.05	0.00	0.00	0.00	Bacteria	Planctomycetes	Pla4 lineage			
0.00	0.00	0.00	0.00	0.02	0.00	0.00	0.00	Bacteria	Planctomycetes	Planctomycetacia	Planctomycetales	Planctomycetaceae	
0.00	0.00	0.00	0.00	0.02	0.00	0.01	0.00	Bacteria	Planctomycetes	Planctomycetacia	Planctomycetales	Planctomycetaceae	Gemmata
0.00	0.00	0.00	0.00	0.05	0.00	0.00	0.00	Bacteria	Planctomycetes	Planctomycetacia	Planctomycetales	Planctomycetaceae	Pir4 lineage
0.00	0.00	0.00	0.00	0.30	0.00	0.01	0.04	Bacteria	Planctomycetes	Planctomycetacia	Planctomycetales	Planctomycetaceae	Pirellula
0.00	0.00	0.00	0.00	1.34	0.02	0.07	0.02	Bacteria	Planctomycetes	Planctomycetacia	Planctomycetales	Planctomycetaceae	Planctomyces
0.00	0.00	0.00	0.00	0.02	0.00	0.00	0.00	Bacteria	Planctomycetes	Planctomycetacia	Planctomycetales	Planctomycetaceae	Rhodopirellula
0.43	0.12	0.00	0.00	0.92	0.10	0.04	0.12	Bacteria	Planctomycetes	Planctomycetacia	Planctomycetales	Planctomycetaceae	Schlesneria
0.00	0.05	0.00	0.00	0.52	0.11	0.01	0.22	Bacteria	Planctomycetes	Planctomycetacia	Planctomycetales	Planctomycetaceae	uncultured
0.00	0.00	0.00	0.00	0.30	0.00	0.01	0.00	Bacteria	Planctomycetes	vadinHA49			
0.00	0.00	0.00	0.00	0.05	0.00	0.00	0.00	Bacteria	Proteobacteria	Alphaproteobacteria	Caulobacterales	Caulobacteraceae	
0.00	0.00	0.00	0.00	0.07	0.00	0.00	0.03	Bacteria	Proteobacteria	Alphaproteobacteria	Caulobacterales	Caulobacteraceae	Brevundimonas
0.00	0.02	0.00	0.00	0.10	0.00	0.02	0.07	Bacteria	Proteobacteria	Alphaproteobacteria	Caulobacterales	Caulobacteraceae	Caulobacter
0.00	0.00	0.00	0.00	0.10	0.06	0.00	0.04	Bacteria	Proteobacteria	Alphaproteobacteria	Caulobacterales	Caulobacteraceae	Phenylobacterium
0.00	0.00	0.01	0.00	0.10	0.02	0.01	0.03	Bacteria	Proteobacteria	Alphaproteobacteria	Caulobacterales	Caulobacteraceae	uncultured
0.00	0.00	0.00	0.00	0.00	0.03	0.00	0.03	Bacteria	Proteobacteria	Alphaproteobacteria	Caulobacterales	Hyphomonadaceae	Hirschia
0.00	0.00	0.00	0.00	0.00	0.02	0.00	0.00	Bacteria	Proteobacteria	Alphaproteobacteria	Caulobacterales	Hyphomonadaceae	uncultured
0.00	0.05	0.00	0.00	0.00	0.06	0.00	0.07	Bacteria	Proteobacteria	Alphaproteobacteria	MNG3		
0.00	0.00	0.00	0.00	0.00	0.00	0.00	0.00	Bacteria	Proteobacteria	Alphaproteobacteria	Rhizobiales	1174-901-12	
0.00	0.00	0.00	0.00	0.02	0.00	0.00	0.00	Bacteria	Proteobacteria	Alphaproteobacteria	Rhizobiales	A0839	
0.00	0.00	0.00	0.00	0.05	0.00	0.00	0.00	Bacteria	Proteobacteria	Alphaproteobacteria	Rhizobiales	Aurantimonadaceae	
0.00	0.00	0.00	0.00	0.05	0.01	0.00	0.03	Bacteria	Proteobacteria	Alphaproteobacteria	Rhizobiales	Beijerinckiaceae	uncultured
0.00	0.00	0.00	0.00	0.00	0.00	0.00	0.02	Bacteria	Proteobacteria	Alphaproteobacteria	Rhizobiales	Bradyrhizobiaceae	Blastobacter
0.00	0.00	0.00	0.01	0.00	0.00	0.00	0.01	Bacteria	Proteobacteria	Alphaproteobacteria	Rhizobiales	Bradyrhizobiaceae	Bosea
0.00	0.00	0.00	0.00	0.07	0.00	0.01	0.02	Bacteria	Proteobacteria	Alphaproteobacteria	Rhizobiales	Bradyrhizobiaceae	Bradyrhizobium
0.00	0.00	0.00	0.00	0.00	0.00	0.00	0.01	Bacteria	Proteobacteria	Alphaproteobacteria	Rhizobiales	Bradyrhizobiaceae	Nitrobacter
0.00	0.00	0.00	0.00	0.00	0.00	0.00	0.01	Bacteria	Proteobacteria	Alphaproteobacteria	Rhizobiales	Bradyrhizobiaceae	Rhodopseudomonas
0.00	0.00	0.00	0.00	0.00	0.00	0.00	0.01	Bacteria	Proteobacteria	Alphaproteobacteria	Rhizobiales	Bradyrhizobiaceae	uncultured
0.00	0.00	0.00	0.00	0.02	0.00	0.00	0.00	Bacteria	Proteobacteria	Alphaproteobacteria	Rhizobiales	Brucellaceae	Ochrobactrum
0.00	0.00	0.00	0.00	0.07	0.00	0.00	0.00	Bacteria	Proteobacteria	Alphaproteobacteria	Rhizobiales	D05-2	
0.00	0.00	0.00	0.00	0.02	0.00	0.00	0.00	Bacteria	Proteobacteria	Alphaproteobacteria	Rhizobiales	DUNssu044	
0.00	0.00	0.00	0.00	0.07	0.02	0.00	0.01	Bacteria	Proteobacteria	Alphaproteobacteria	Rhizobiales	DUNssu371	
0.00	0.05	0.00	0.00	0.00	0.00	0.00	0.01	Bacteria	Proteobacteria	Alphaproteobacteria	Rhizobiales	F0723	
0.00	0.00	0.00	0.00	0.00	0.00	0.01	0.00	Bacteria	Proteobacteria	Alphaproteobacteria	Rhizobiales	Family Incertae Sedis	Agaricicola
0.00	0.00	0.00	0.00	0.02	0.00	0.00	0.00	Bacteria	Proteobacteria	Alphaproteobacteria	Rhizobiales	Family Incertae Sedis	Bauldia
0.00	0.00	0.00	0.00	0.00	0.00	0.00	0.01	Bacteria	Proteobacteria	Alphaproteobacteria	Rhizobiales	Family Incertae Sedis	Rhizomicrobium
0.00	0.00	0.00	0.00	0.00	0.02	0.00	0.01	Bacteria	Proteobacteria	Alphaproteobacteria	Rhizobiales	FukuN57	
0.00	0.00	0.00	0.00	0.00	0.00	0.01	0.01	Bacteria	Proteobacteria	Alphaproteobacteria	Rhizobiales	Hyphomicrobiaceae	Blastochloris
0.00	0.00	0.00	0.00	0.02	0.01	0.01	0.02	Bacteria	Proteobacteria	Alphaproteobacteria	Rhizobiales	Hyphomicrobiaceae	Devosia
0.00	0.00	0.00	0.00	0.00	0.00	0.00	0.00	Bacteria	Proteobacteria	Alphaproteobacteria	Rhizobiales	Hyphomicrobiaceae	Filomicrobium
0.86	0.02	0.00	0.00	0.30	0.01	0.02	0.01	Bacteria	Proteobacteria	Alphaproteobacteria	Rhizobiales	Hyphomicrobiaceae	Hyphomicrobium
0.00	0.00	0.00	0.00	0.17	0.00	0.02	0.00	Bacteria	Proteobacteria	Alphaproteobacteria	Rhizobiales	Hyphomicrobiaceae	Pedomicrobium
0.00	0.00	0.00	0.00	0.00	0.02	0.00	0.07	Bacteria	Proteobacteria	Alphaproteobacteria	Rhizobiales	Hyphomicrobiaceae	Rhodoplanes
0.00	0.00	0.00	0.00	0.00	0.00	0.00	0.03	Bacteria	Proteobacteria	Alphaproteobacteria	Rhizobiales	JG34-KF-361	
0.00	0.05	0.00	0.00	0.00	0.03	0.00	0.06	Bacteria	Proteobacteria	Alphaproteobacteria	Rhizobiales	KF-JG30-B3	
0.43	0.02	0.00	0.00	0.32	0.05	0.01	0.02	Bacteria	Proteobacteria	Alphaproteobacteria	Rhizobiales	MNG7	
0.43	0.00	0.00	0.00	0.22	0.00	0.02	0.02	Bacteria	Proteobacteria	Alphaproteobacteria	Rhizobiales	Methylobacteriaceae	Methylobacterium
0.00	0.00	0.03	0.00	0.00	0.00	0.00	0.00	Bacteria	Proteobacteria	Alphaproteobacteria	Rhizobiales	Methylobacteriaceae	uncultured

Phase(I)-EL-PA	Phase(I)-EL-FL	Phase(II)-EL-PA	Phase(II)-EL-FL	Phase(I)-HL-PA	Phase(I)-HL-FL	Phase(II)-HL-PA	Phase(II)-HL-FL	Domain	Phylum	Class	Order	Family	Genus
0.00	0.00	0.00	0.00	0.05	0.00	0.00	0.00	Bacteria	Proteobacteria	Alphaproteobacteria	Rhizobiales	Methylocystaceae	Methylosinus
0.00	0.02	0.00	0.00	0.00	0.04	0.00	0.01	Bacteria	Proteobacteria	Alphaproteobacteria	Rhizobiales	Methylocystaceae	uncultured
0.00	0.00	0.00	0.00	0.00	0.00	0.00	0.00	Bacteria	Proteobacteria	Alphaproteobacteria	Rhizobiales	Phyllobacteriaceae	Mesorhizobium
0.00	0.00	0.00	0.00	0.02	0.00	0.00	0.00	Bacteria	Proteobacteria	Alphaproteobacteria	Rhizobiales	Phyllobacteriaceae	uncultured
0.00	0.00	0.00	0.00	0.02	0.00	0.01	0.01	Bacteria	Proteobacteria	Alphaproteobacteria	Rhizobiales	Rhizobiaceae	Rhizobium
0.86	0.00	0.00	0.00	0.12	0.00	0.00	0.00	Bacteria	Proteobacteria	Alphaproteobacteria	Rhizobiales	Rhodobiaceae	Rhodobium
0.00	0.00	0.00	0.00	0.00	0.02	0.00	0.00	Bacteria	Proteobacteria	Alphaproteobacteria	Rhizobiales	Xanthobacteraceae	Labrys
0.00	0.00	0.00	0.00	0.02	0.00	0.00	0.00	Bacteria	Proteobacteria	Alphaproteobacteria	Rhizobiales	Xanthobacteraceae	Pseudolabrys
0.00	0.00	0.00	0.00	0.07	0.00	0.00	0.00	Bacteria	Proteobacteria	Alphaproteobacteria	Rhizobiales	Xanthobacteraceae	Pseudoxanthobacter
0.00	0.00	0.00	0.00	0.02	0.02	0.00	0.02	Bacteria	Proteobacteria	Alphaproteobacteria	Rhizobiales	Xanthobacteraceae	uncultured
0.00	0.10	0.00	0.00	0.00	0.11	0.00	0.19	Bacteria	Proteobacteria	Alphaproteobacteria	Rhizobiales	alphaI cluster	
0.43	0.00	0.00	0.00	0.22	0.00	0.01	0.00	Bacteria	Proteobacteria	Alphaproteobacteria	Rhodobacterales	Rhodobacteraceae	Paracoccus
0.00	0.00	0.00	0.00	0.07	0.00	0.00	0.00	Bacteria	Proteobacteria	Alphaproteobacteria	Rhodobacterales	Rhodobacteraceae	Pseudorhodobacter
0.00	0.02	0.00	0.00	0.10	0.14	0.01	0.14	Bacteria	Proteobacteria	Alphaproteobacteria	Rhodobacterales	Rhodobacteraceae	Rhodobacter
0.00	0.00	0.00	0.00	0.00	0.00	0.01	0.00	Bacteria	Proteobacteria	Alphaproteobacteria	Rhodobacterales	Rhodobacteraceae	Rhodovulum
0.00	0.00	0.00	0.00	0.00	0.00	0.00	0.00	Bacteria	Proteobacteria	Alphaproteobacteria	Rhodobacterales	Rhodobacteraceae	Roseovarius
0.00	0.00	0.00	0.00	0.07	0.00	0.00	0.00	Bacteria	Proteobacteria	Alphaproteobacteria	Rhodobacterales	Rhodobacteraceae	Rubellimicrobium
0.00	0.00	0.00	0.00	0.07	0.00	0.00	0.00	Bacteria	Proteobacteria	Alphaproteobacteria	Rhodobacterales	Rhodobacteraceae	Ruegeria
0.00	0.00	0.00	0.00	0.22	0.00	0.02	0.00	Bacteria	Proteobacteria	Alphaproteobacteria	Rhodobacterales	Rhodobacteraceae	uncultured
0.00	0.00	0.00	0.00	0.00	0.02	0.01	0.02	Bacteria	Proteobacteria	Alphaproteobacteria	Rhodospirillales	Acetobacteraceae	
0.00	0.00	0.00	0.00	0.00	0.01	0.00	0.00	Bacteria	Proteobacteria	Alphaproteobacteria	Rhodospirillales	Acetobacteraceae	Acidiphilium
0.00	0.00	0.00	0.00	0.00	0.01	0.00	0.00	Bacteria	Proteobacteria	Alphaproteobacteria	Rhodospirillales	Acetobacteraceae	Acidocella
0.00	0.05	0.00	0.00	0.05	0.07	0.00	0.04	Bacteria	Proteobacteria	Alphaproteobacteria	Rhodospirillales	Acetobacteraceae	Rhodovastum
0.00	0.00	0.00	0.01	0.45	0.00	0.01	0.03	Bacteria	Proteobacteria	Alphaproteobacteria	Rhodospirillales	Acetobacteraceae	Roseomonas
0.00	0.02	0.00	0.00	0.00	0.02	0.01	0.01	Bacteria	Proteobacteria	Alphaproteobacteria	Rhodospirillales	Acetobacteraceae	uncultured
0.00	0.00	0.00	0.00	0.00	0.01	0.00	0.00	Bacteria	Proteobacteria	Alphaproteobacteria	Rhodospirillales	DA111	
0.00	0.05	0.00	0.00	0.15	0.01	0.00	0.02	Bacteria	Proteobacteria	Alphaproteobacteria	Rhodospirillales	I-10	
0.00	0.00	0.00	0.00	0.12	0.00	0.01	0.02	Bacteria	Proteobacteria	Alphaproteobacteria	Rhodospirillales	JG37-AG-20	
0.00	0.00	0.00	0.00	0.02	0.01	0.01	0.01	Bacteria	Proteobacteria	Alphaproteobacteria	Rhodospirillales	Rhodospirillaceae	Azospirillum
0.00	0.00	0.00	0.01	0.05	0.03	0.00	0.16	Bacteria	Proteobacteria	Alphaproteobacteria	Rhodospirillales	Rhodospirillaceae	Defluviicoccus
0.00	0.00	0.00	0.00	0.10	0.00	0.00	0.01	Bacteria	Proteobacteria	Alphaproteobacteria	Rhodospirillales	Rhodospirillaceae	Skermanella
0.00	0.07	0.00	0.00	0.42	0.11	0.00	0.26	Bacteria	Proteobacteria	Alphaproteobacteria	Rhodospirillales	Rhodospirillaceae	uncultured
0.00	0.10	0.00	0.00	0.32	0.16	0.01	0.20	Bacteria	Proteobacteria	Alphaproteobacteria	Rhodospirillales	wr0007	
0.00	0.00	0.00	0.00	0.00	0.00	0.00	0.01	Bacteria	Proteobacteria	Alphaproteobacteria	Rickettsiales		
0.00	0.02	0.00	0.00	0.57	0.03	0.02	0.04	Bacteria	Proteobacteria	Alphaproteobacteria	Rickettsiales	Candidatus Captivus	
0.00	0.00	0.00	0.00	0.05	0.00	0.00	0.00	Bacteria	Proteobacteria	Alphaproteobacteria	Rickettsiales	EF100-94H03	
0.00	0.00	0.00	0.00	0.05	0.00	0.00	0.00	Bacteria	Proteobacteria	Alphaproteobacteria	Rickettsiales	Ho(lab)	
0.00	0.05	0.00	0.00	0.00	0.00	0.00	0.00	Bacteria	Proteobacteria	Alphaproteobacteria	Rickettsiales	Rickettsiaceae	Candidatus Cryptoprodotis
0.86	0.00	0.01	0.00	0.70	0.01	0.04	0.00	Bacteria	Proteobacteria	Alphaproteobacteria	Rickettsiales	Rickettsiaceae	Rickettsia
0.00	0.00	0.00	0.00	0.00	0.00	0.01	0.00	Bacteria	Proteobacteria	Alphaproteobacteria	Rickettsiales	SAR116 clade	
0.00	0.00	0.00	0.00	0.00	0.01	0.00	0.00	Bacteria	Proteobacteria	Alphaproteobacteria	Rickettsiales	SM2D12	
0.00	0.00	0.00	0.00	0.00	0.02	0.00	0.01	Bacteria	Proteobacteria	Alphaproteobacteria	Rickettsiales	TK34	
1.72	0.00	0.01	0.00	0.40	0.08	0.02	0.07	Bacteria	Proteobacteria	Alphaproteobacteria	Rickettsiales	mitochondria	
0.00	0.00	0.00	0.00	0.02	0.00	0.00	0.00	Bacteria	Proteobacteria	Alphaproteobacteria	Rickettsiales	uncultured	
0.00	0.22	0.00	0.01	0.15	0.13	0.01	0.17	Bacteria	Proteobacteria	Alphaproteobacteria	SAR11 clade		
1.29	23.88	0.01	0.20	1.17	21.92	0.01	11.77	Bacteria	Proteobacteria	Alphaproteobacteria	SAR11 clade	LD12 freshwater group	
0.00	0.07	0.00	0.00	0.00	1.29	0.00	0.14	Bacteria	Proteobacteria	Alphaproteobacteria	SAR11 clade	Surface 1	
0.00	0.00	0.00	0.00	0.10	0.00	0.00	0.00	Bacteria	Proteobacteria	Alphaproteobacteria	Sphingomonadales	Ellin6055	
0.43	0.00	0.00	0.00	0.15	0.00	0.00	0.00	Bacteria	Proteobacteria	Alphaproteobacteria	Sphingomonadales	Erythrobacteraceae	Erythrobacter
12.88	0.05	0.00	0.00	2.71	0.03	0.02	0.04	Bacteria	Proteobacteria	Alphaproteobacteria	Sphingomonadales	GOBB3-C201	
0.00	0.05	0.00	0.00	0.00	0.01	0.00	0.00	Bacteria	Proteobacteria	Alphaproteobacteria	Sphingomonadales	M05-Pitesti	
0.00	0.02	0.00	0.00	0.22	0.01	0.00	0.06	Bacteria	Proteobacteria	Alphaproteobacteria	Sphingomonadales	Sphingomonadaceae	Novosphingobium

Phase(I)-EL-PA	Phase(I)-EL-FL	Phase(II)-EL-PA	Phase(II)-EL-FL	Phase(I)-HL-PA	Phase(I)-HL-FL	Phase(II)-HL-PA	Phase(II)-HL-FL	Domain	Phylum	Class	Order	Family	Genus
0.00	0.00	0.00	0.00	0.10	0.00	0.00	0.00	Bacteria	Proteobacteria	Alphaproteobacteria	Sphingomonadales	Sphingomonadaceae	Sphingobium
0.43	0.00	0.00	0.00	0.17	0.03	0.02	0.05	Bacteria	Proteobacteria	Alphaproteobacteria	Sphingomonadales	Sphingomonadaceae	Sphingomonas
0.00	0.00	0.00	0.00	0.02	0.00	0.00	0.00	Bacteria	Proteobacteria	Alphaproteobacteria	Sphingomonadales	Sphingomonadaceae	Sphingopyxis
0.00	0.00	0.00	0.00	0.05	0.00	0.00	0.00	Bacteria	Proteobacteria	Alphaproteobacteria	Sphingomonadales	Sphingomonadaceae	Zymomonas
0.00	0.00	0.00	0.00	0.35	0.00	0.00	0.00	Bacteria	Proteobacteria	Betaproteobacteria	B1-7BS		
1.29	0.00	0.00	0.00	0.07	0.05	0.00	0.14	Bacteria	Proteobacteria	Betaproteobacteria	Burkholderiales	Alcaligenaceae	Achromobacter
0.00	0.00	0.00	0.00	0.00	0.01	0.00	0.01	Bacteria	Proteobacteria	Betaproteobacteria	Burkholderiales	Alcaligenaceae	Alcaligenes
0.00	0.02	0.00	0.00	0.07	0.03	0.00	0.01	Bacteria	Proteobacteria	Betaproteobacteria	Burkholderiales	Alcaligenaceae	Derxia
0.00	0.10	0.01	0.01	0.07	0.20	0.00	0.10	Bacteria	Proteobacteria	Betaproteobacteria	Burkholderiales	Alcaligenaceae	GKS98 freshwater group
0.00	0.02	0.00	0.00	0.00	0.00	0.00	0.00	Bacteria	Proteobacteria	Betaproteobacteria	Burkholderiales	Alcaligenaceae	Kerstersia
0.00	0.00	0.00	0.00	0.07	0.00	0.00	0.00	Bacteria	Proteobacteria	Betaproteobacteria	Burkholderiales	Alcaligenaceae	MWH-UniP1 aquatic group
0.00	0.00	0.01	0.00	0.42	0.00	0.00	0.01	Bacteria	Proteobacteria	Betaproteobacteria	Burkholderiales	Alcaligenaceae	uncultured
0.00	0.00	0.00	0.00	0.00	0.01	0.00	0.01	Bacteria	Proteobacteria	Betaproteobacteria	Burkholderiales	Burkholderiaceae	Limnobacter
0.00	0.29	0.45	0.95	0.52	0.44	0.18	0.52	Bacteria	Proteobacteria	Betaproteobacteria	Burkholderiales	Burkholderiaceae	Polynucleobacter
0.00	0.00	0.00	0.00	0.02	0.01	0.00	0.00	Bacteria	Proteobacteria	Betaproteobacteria	Burkholderiales	Burkholderiaceae	Ralstonia
0.00	0.00	0.00	0.00	0.02	0.01	0.00	0.04	Bacteria	Proteobacteria	Betaproteobacteria	Burkholderiales	CM1G08	
0.00	0.05	0.01	0.00	0.00	0.05	0.01	0.02	Bacteria	Proteobacteria	Betaproteobacteria	Burkholderiales	Comamonadaceae	
0.00	0.02	0.00	0.00	0.05	0.00	0.00	0.03	Bacteria	Proteobacteria	Betaproteobacteria	Burkholderiales	Comamonadaceae	Acidovorax
3.86	0.36	2.79	10.69	0.45	0.50	2.51	1.36	Bacteria	Proteobacteria	Betaproteobacteria	Burkholderiales	Comamonadaceae	Albidiferax
0.00	0.00	0.01	0.00	0.00	0.00	0.00	0.00	Bacteria	Proteobacteria	Betaproteobacteria	Burkholderiales	Comamonadaceae	Aquabacterium
0.00	0.00	0.00	0.00	0.00	0.02	0.00	0.01	Bacteria	Proteobacteria	Betaproteobacteria	Burkholderiales	Comamonadaceae	BAL58 marine group
0.00	0.00	0.00	0.00	0.00	0.01	0.00	0.04	Bacteria	Proteobacteria	Betaproteobacteria	Burkholderiales	Comamonadaceae	Caenimonas
0.00	0.56	0.53	18.01	0.02	0.16	0.19	3.60	Bacteria	Proteobacteria	Betaproteobacteria	Burkholderiales	Comamonadaceae	Chlorochromatium
0.00	0.00	0.00	0.00	0.07	0.00	0.00	0.00	Bacteria	Proteobacteria	Betaproteobacteria	Burkholderiales	Comamonadaceae	Comamonas
0.00	0.00	0.00	0.00	0.02	0.02	0.00	0.01	Bacteria	Proteobacteria	Betaproteobacteria	Burkholderiales	Comamonadaceae	Hydrogenophaga
0.00	0.00	0.00	0.00	0.05	0.00	0.00	0.00	Bacteria	Proteobacteria	Betaproteobacteria	Burkholderiales	Comamonadaceae	Leptothrix
4.29	1.38	0.38	0.14	0.52	1.09	0.25	0.62	Bacteria	Proteobacteria	Betaproteobacteria	Burkholderiales	Comamonadaceae	Limnohabitans
0.00	0.00	0.00	0.01	0.00	0.00	0.00	0.00	Bacteria	Proteobacteria	Betaproteobacteria	Burkholderiales	Comamonadaceae	Malikia
0.00	0.00	0.00	0.00	0.02	0.01	0.00	0.00	Bacteria	Proteobacteria	Betaproteobacteria	Burkholderiales	Comamonadaceae	Methylibium
0.00	0.00	0.42	0.07	0.00	0.00	0.39	0.07	Bacteria	Proteobacteria	Betaproteobacteria	Burkholderiales	Comamonadaceae	Paucibacter
0.00	0.00	0.07	0.00	0.05	0.00	0.00	0.00	Bacteria	Proteobacteria	Betaproteobacteria	Burkholderiales	Comamonadaceae	Pelomonas
0.00	0.00	0.00	0.00	0.02	0.00	0.00	0.00	Bacteria	Proteobacteria	Betaproteobacteria	Burkholderiales	Comamonadaceae	Piscinibacter
12.02	0.10	1.22	0.03	2.19	0.11	0.01	0.09	Bacteria	Proteobacteria	Betaproteobacteria	Burkholderiales	Comamonadaceae	Polaromonas
0.00	0.00	0.00	0.00	0.00	0.00	0.00	0.02	Bacteria	Proteobacteria	Betaproteobacteria	Burkholderiales	Comamonadaceae	Pseudorhodoferax
0.00	0.02	0.00	0.00	0.00	0.08	0.00	0.04	Bacteria	Proteobacteria	Betaproteobacteria	Burkholderiales	Comamonadaceae	Ramlibacter
0.00	0.00	0.00	0.00	0.22	0.00	0.00	0.00	Bacteria	Proteobacteria	Betaproteobacteria	Burkholderiales	Comamonadaceae	Rhizobacter
0.00	0.00	0.00	0.00	0.12	0.00	0.00	0.00	Bacteria	Proteobacteria	Betaproteobacteria	Burkholderiales	Comamonadaceae	Rubrivivax
0.00	0.00	0.00	0.02	0.37	0.02	0.00	0.04	Bacteria	Proteobacteria	Betaproteobacteria	Burkholderiales	Comamonadaceae	Simplicispira
0.00	0.12	0.00	0.01	0.02	0.02	0.01	0.02	Bacteria	Proteobacteria	Betaproteobacteria	Burkholderiales	Comamonadaceae	Variovorax
0.00	0.00	0.00	0.00	0.00	0.01	0.00	0.00	Bacteria	Proteobacteria	Betaproteobacteria	Burkholderiales	Comamonadaceae	Xenophilus
0.43	1.09	21.64	9.45	1.07	0.62	8.97	2.53	Bacteria	Proteobacteria	Betaproteobacteria	Burkholderiales	Comamonadaceae	uncultured
0.00	0.00	2.11	0.75	0.02	0.01	2.74	0.17	Bacteria	Proteobacteria	Betaproteobacteria	Burkholderiales	Oxalobacteraceae	Aquaspirillum
0.00	0.00	0.05	0.00	0.00	0.00	0.00	0.00	Bacteria	Proteobacteria	Betaproteobacteria	Burkholderiales	Oxalobacteraceae	Duganella
0.00	0.00	0.01	0.01	0.00	0.00	0.00	0.00	Bacteria	Proteobacteria	Betaproteobacteria	Burkholderiales	Oxalobacteraceae	Herminiimonas
0.00	0.00	0.09	0.00	0.00	0.00	0.07	0.00	Bacteria	Proteobacteria	Betaproteobacteria	Burkholderiales	Oxalobacteraceae	Janthinobacterium
0.00	0.07	11.01	0.62	2.29	0.03	5.31	0.11	Bacteria	Proteobacteria	Betaproteobacteria	Burkholderiales	Oxalobacteraceae	Massilia
0.00	0.02	0.00	0.00	0.00	0.02	0.00	0.06	Bacteria	Proteobacteria	Betaproteobacteria	Burkholderiales	Oxalobacteraceae	Paucimonas
0.43	0.00	35.32	0.91	0.12	0.00	60.22	0.48	Bacteria	Proteobacteria	Betaproteobacteria	Burkholderiales	Oxalobacteraceae	Undibacterium
0.00	0.02	0.00	0.00	0.00	0.00	0.02	0.00	Bacteria	Proteobacteria	Betaproteobacteria	Burkholderiales	Oxalobacteraceae	uncultured
0.00	0.00	0.00	0.00	0.05	0.00	0.00	0.01	Bacteria	Proteobacteria	Betaproteobacteria	Hot Creek 32		
0.00	0.00	0.00	0.00	0.02	0.00	0.00	0.00	Bacteria	Proteobacteria	Betaproteobacteria	Hydrogenophilales	Hydrogenophilaceae	Ferritrophicum
0.00	0.00	0.00	0.00	0.12	0.00	0.00	0.00	Bacteria	Proteobacteria	Betaproteobacteria	Hydrogenophilales	Hydrogenophilaceae	Sulfuricella

Phase(I)-EL-PA	Phase(I)-EL-FL	Phase(II)-EL-PA	Phase(II)-EL-FL	Phase(I)-HL-PA	Phase(I)-HL-FL	Phase(II)-HL-PA	Phase(II)-HL-FL	Domain	Phylum	Class	Order	Family	Genus
0.00	0.73	0.00	0.07	0.47	0.47	0.01	0.52	Bacteria	Proteobacteria	Betaproteobacteria	Hydrogenophilales	Hydrogenophilaceae	Thiobacillus
0.00	0.00	0.00	0.00	0.25	0.00	0.01	0.00	Bacteria	Proteobacteria	Betaproteobacteria	Hydrogenophilales	Hydrogenophilaceae	uncultured
0.00	0.00	0.00	0.00	0.00	0.06	0.00	0.02	Bacteria	Proteobacteria	Betaproteobacteria	Methylophilales	Methylophilaceae	LD28 freshwater group
0.00	0.00	0.05	0.00	0.00	0.00	0.02	0.02	Bacteria	Proteobacteria	Betaproteobacteria	Methylophilales	Methylophilaceae	Methylotenera
0.86	1.29	0.12	0.07	0.35	1.63	0.09	1.56	Bacteria	Proteobacteria	Betaproteobacteria	Methylophilales	Methylophilaceae	OM43 clade
0.00	0.00	0.04	0.00	0.00	0.00	0.00	0.00	Bacteria	Proteobacteria	Betaproteobacteria	Neisseriales	Neisseriaceae	Iodobacter
0.43	0.00	0.00	0.00	0.12	0.01	0.00	0.00	Bacteria	Proteobacteria	Betaproteobacteria	Neisseriales	Neisseriaceae	Leeia
0.43	0.00	0.00	0.00	0.22	0.01	0.00	0.01	Bacteria	Proteobacteria	Betaproteobacteria	Neisseriales	Neisseriaceae	Vogesella
0.00	0.02	0.00	0.00	0.00	0.00	0.00	0.00	Bacteria	Proteobacteria	Betaproteobacteria	Neisseriales	Neisseriaceae	uncultured
0.00	0.00	0.00	0.00	0.00	0.11	0.00	0.08	Bacteria	Proteobacteria	Betaproteobacteria	Nitrosomonadales	Gallionellaceae	Candidatus Nitrotoga
0.00	0.00	0.00	0.00	0.10	0.00	0.00	0.00	Bacteria	Proteobacteria	Betaproteobacteria	Nitrosomonadales	Gallionellaceae	Sideroxydans
0.00	0.00	0.00	0.00	0.05	0.00	0.01	0.00	Bacteria	Proteobacteria	Betaproteobacteria	Nitrosomonadales	Gallionellaceae	uncultured
0.00	0.34	0.00	0.01	0.05	1.94	0.00	2.56	Bacteria	Proteobacteria	Betaproteobacteria	Nitrosomonadales	Nitrosomonadaceae	Nitrosomonas
0.00	0.00	0.00	0.00	0.00	0.01	0.00	0.03	Bacteria	Proteobacteria	Betaproteobacteria	Nitrosomonadales	Nitrosomonadaceae	Nitrosospira
3.01	0.12	0.00	0.01	1.44	0.15	0.03	0.20	Bacteria	Proteobacteria	Betaproteobacteria	Nitrosomonadales	Nitrosomonadaceae	uncultured
0.00	0.05	0.00	0.00	0.02	0.01	0.00	0.04	Bacteria	Proteobacteria	Betaproteobacteria	Rhodocyclales	Rhodocyclaceae	Azospira
0.00	0.00	0.00	0.00	0.17	0.01	0.01	0.00	Bacteria	Proteobacteria	Betaproteobacteria	Rhodocyclales	Rhodocyclaceae	Dechloromonas
0.86	0.00	0.00	0.00	0.00	0.00	0.00	0.01	Bacteria	Proteobacteria	Betaproteobacteria	Rhodocyclales	Rhodocyclaceae	Georgfuchsia
0.00	0.00	0.00	0.00	0.02	0.00	0.00	0.00	Bacteria	Proteobacteria	Betaproteobacteria	Rhodocyclales	Rhodocyclaceae	Methyloversatilis
0.00	0.00	0.00	0.00	0.20	0.01	0.00	0.03	Bacteria	Proteobacteria	Betaproteobacteria	Rhodocyclales	Rhodocyclaceae	Propionivibrio
0.00	0.00	0.00	0.00	0.02	0.00	0.01	0.01	Bacteria	Proteobacteria	Betaproteobacteria	Rhodocyclales	Rhodocyclaceae	Quatrionicoccus
0.00	0.05	0.00	0.00	0.47	0.03	0.00	0.02	Bacteria	Proteobacteria	Betaproteobacteria	Rhodocyclales	Rhodocyclaceae	Sulfuritalea
0.00	0.00	0.00	0.00	0.05	0.00	0.00	0.00	Bacteria	Proteobacteria	Betaproteobacteria	Rhodocyclales	Rhodocyclaceae	Uliginosibacterium
0.00	0.00	0.00	0.00	0.00	0.00	0.01	0.00	Bacteria	Proteobacteria	Betaproteobacteria	Rhodocyclales	Rhodocyclaceae	Zoogloea
0.00	0.05	0.00	0.02	0.02	0.04	0.02	0.04	Bacteria	Proteobacteria	Betaproteobacteria	Rhodocyclales	Rhodocyclaceae	uncultured
0.00	0.00	0.03	0.00	0.70	0.00	0.01	0.01	Bacteria	Proteobacteria	Betaproteobacteria	SC-I-84		
0.00	0.78	0.00	0.29	0.27	0.24	0.04	0.34	Bacteria	Proteobacteria	Betaproteobacteria	TRA3-20		
0.00	0.00	0.00	0.00	0.10	0.00	0.00	0.00	Bacteria	Proteobacteria	CF2			
0.00	0.00	0.00	0.00	0.07	0.00	0.00	0.00	Bacteria	Proteobacteria	Deltaproteobacteria	43F-1404R		
0.00	0.07	0.00	0.00	0.00	0.01	0.00	0.06	Bacteria	Proteobacteria	Deltaproteobacteria	Bdellovibrionales	Bacteriovoracaceae	Peredibacter
0.00	0.00	0.00	0.00	0.15	0.00	0.00	0.00	Bacteria	Proteobacteria	Deltaproteobacteria	Bdellovibrionales	Bacteriovoracaceae	uncultured
0.00	0.00	0.00	0.00	0.07	0.00	0.00	0.00	Bacteria	Proteobacteria	Deltaproteobacteria	Bdellovibrionales	Bdellovibrionaceae	Bdellovibrio
0.43	0.00	0.00	0.00	0.37	0.05	0.00	0.02	Bacteria	Proteobacteria	Deltaproteobacteria	Bdellovibrionales	Bdellovibrionaceae	OM27 clade
0.00	0.00	0.00	0.00	0.50	0.00	0.00	0.00	Bacteria	Proteobacteria	Deltaproteobacteria	Desulfarculales	Desulfarculaceae	uncultured
0.00	0.00	0.00	0.00	0.07	0.00	0.00	0.00	Bacteria	Proteobacteria	Deltaproteobacteria	Desulfobacterales	Desulfobacteraceae	Desulfatirhabdium
0.00	0.00	0.00	0.00	0.02	0.00	0.00	0.00	Bacteria	Proteobacteria	Deltaproteobacteria	Desulfobacterales	Desulfobacteraceae	Desulfobacterium
0.00	0.00	0.00	0.00	0.07	0.00	0.00	0.00	Bacteria	Proteobacteria	Deltaproteobacteria	Desulfobacterales	Desulfobacteraceae	SEEP-SRB1
0.00	0.00	0.00	0.00	0.52	0.00	0.00	0.00	Bacteria	Proteobacteria	Deltaproteobacteria	Desulfobacterales	Desulfobacteraceae	uncultured
0.00	0.00	0.00	0.00	0.02	0.00	0.00	0.00	Bacteria	Proteobacteria	Deltaproteobacteria	Desulfobacterales	Desulfobulbaceae	uncultured
0.00	0.00	0.00	0.00	0.17	0.00	0.01	0.00	Bacteria	Proteobacteria	Deltaproteobacteria	Desulfobacterales	Nitrospinaceae	uncultured
0.00	0.00	0.00	0.00	0.02	0.00	0.00	0.00	Bacteria	Proteobacteria	Deltaproteobacteria	Desulfuromonadales	BVA18	
0.43	0.00	0.00	0.00	0.27	0.00	0.01	0.02	Bacteria	Proteobacteria	Deltaproteobacteria	Desulfuromonadales	GR-WP33-58	
0.43	0.00	0.00	0.00	0.62	0.01	0.01	0.00	Bacteria	Proteobacteria	Deltaproteobacteria	GR-WP33-30		
0.00	0.00	0.00	0.00	0.17	0.02	0.01	0.01	Bacteria	Proteobacteria	Deltaproteobacteria	Myxococcales	0319-6G20	
0.00	0.00	0.00	0.00	0.02	0.00	0.00	0.00	Bacteria	Proteobacteria	Deltaproteobacteria	Myxococcales	Cystobacterineae	Cystobacteraceae
0.00	0.00	0.00	0.00	0.02	0.00	0.00	0.00	Bacteria	Proteobacteria	Deltaproteobacteria	Myxococcales	Cystobacterineae	uncultured
0.00	0.00	0.00	0.00	0.05	0.00	0.00	0.00	Bacteria	Proteobacteria	Deltaproteobacteria	Myxococcales	Elev-16S-1158	
0.00	0.00	0.00	0.00	1.20	0.00	0.00	0.00	Bacteria	Proteobacteria	Deltaproteobacteria	Myxococcales	Nannocystineae	Nannocystaceae
0.43	0.00	0.00	0.00	0.10	0.00	0.00	0.01	Bacteria	Proteobacteria	Deltaproteobacteria	Myxococcales	Nannocystineae	uncultured
0.00	0.00	0.00	0.00	0.00	0.00	0.00	0.01	Bacteria	Proteobacteria	Deltaproteobacteria	Myxococcales	Sorangiineae	Phaselicystidaceae
0.00	0.00	0.00	0.00	0.02	0.03	0.00	0.00	Bacteria	Proteobacteria	Deltaproteobacteria	Myxococcales	Sorangiineae	Polyangiaceae
0.00	0.00	0.00	0.00	0.02	0.00	0.00	0.00	Bacteria	Proteobacteria	Deltaproteobacteria	Myxococcales	Sorangiineae	Sandaracinaceae

Phase(I)-EL-PA	Phase(I)-EL-FL	Phase(II)-EL-PA	Phase(II)-EL-FL	Phase(I)-HL-PA	Phase(I)-HL-FL	Phase(II)-HL-PA	Phase(II)-HL-FL	Domain	Phylum	Class	Order	Family	Genus
0.00	0.02	0.00	0.00	0.10	0.00	0.00	0.00	Bacteria	Proteobacteria	Deltaproteobacteria	Myxococcales	Sorangiineae	uncultured
0.00	0.00	0.00	0.00	0.02	0.00	0.00	0.01	Bacteria	Proteobacteria	Deltaproteobacteria	Myxococcales	mle1-27	
0.00	0.00	0.00	0.00	0.02	0.00	0.00	0.00	Bacteria	Proteobacteria	Deltaproteobacteria	Order Incertae Sedis	Syntrophorhabdaceae	Syntrophorhabdus
0.00	0.00	0.00	0.00	0.40	0.00	0.00	0.01	Bacteria	Proteobacteria	Deltaproteobacteria	Sh765B-TzT-29		
0.00	0.00	0.00	0.00	1.69	0.00	0.00	0.00	Bacteria	Proteobacteria	Deltaproteobacteria	Sva0485		
0.00	0.00	0.00	0.00	0.17	0.00	0.00	0.00	Bacteria	Proteobacteria	Deltaproteobacteria	Syntrophobacterales	Syntrophaceae	Desulfobacca
0.00	0.19	0.00	0.00	0.00	0.05	0.00	0.03	Bacteria	Proteobacteria	Deltaproteobacteria	Syntrophobacterales	Syntrophaceae	Smithella
0.00	0.00	0.00	0.00	0.20	0.00	0.00	0.00	Bacteria	Proteobacteria	Deltaproteobacteria	Syntrophobacterales	Syntrophaceae	Syntrophus
0.00	0.00	0.00	0.00	0.40	0.00	0.00	0.00	Bacteria	Proteobacteria	Deltaproteobacteria	Syntrophobacterales	Syntrophaceae	uncultured
0.00	0.00	0.00	0.00	0.05	0.00	0.00	0.00	Bacteria	Proteobacteria	Deltaproteobacteria	Syntrophobacterales	Syntrophobacteraceae	Desulfovirga
0.00	0.00	0.00	0.00	0.30	0.00	0.00	0.01	Bacteria	Proteobacteria	Deltaproteobacteria	Syntrophobacterales	Syntrophobacteraceae	uncultured
0.00	0.00	0.00	0.00	0.17	0.00	0.00	0.01	Bacteria	Proteobacteria	Elev-16S-509			
0.43	0.02	0.00	0.00	0.10	0.03	0.01	0.01	Bacteria	Proteobacteria	Epsilonproteobacteria	Campylobacterales	Campylobacteraceae	Arcobacter
0.00	0.00	0.00	0.00	0.12	0.00	0.00	0.02	Bacteria	Proteobacteria	Epsilonproteobacteria	Campylobacterales	Helicobacteraceae	Sulfuricurvum
0.00	0.00	0.00	0.00	0.05	0.00	0.00	0.00	Bacteria	Proteobacteria	Epsilonproteobacteria	Campylobacterales	Helicobacteraceae	Sulfurimonas
0.43	0.00	0.00	0.01	0.00	0.00	0.01	0.02	Bacteria	Proteobacteria	Epsilonproteobacteria	Campylobacterales	Helicobacteraceae	Sulfurovum
0.00	0.00	0.00	0.00	0.00	0.00	0.00	0.01	Bacteria	Proteobacteria	Gammaproteobacteria	1013-28-CG33		
0.00	0.19	0.00	0.01	0.02	0.68	0.00	0.92	Bacteria	Proteobacteria	Gammaproteobacteria	Acidithiobacillales	KCM-B-112	
0.00	0.05	0.00	0.00	0.00	0.02	0.00	0.05	Bacteria	Proteobacteria	Gammaproteobacteria	Alteromonadales	Alteromonadaceae	BD1-7 clade
0.00	0.00	0.00	0.00	0.05	0.00	0.00	0.00	Bacteria	Proteobacteria	Gammaproteobacteria	Alteromonadales	Alteromonadaceae	C1-B045
0.00	0.00	0.00	0.00	0.00	0.00	0.00	0.00	Bacteria	Proteobacteria	Gammaproteobacteria	Alteromonadales	Alteromonadaceae	Dasania
0.00	0.00	0.00	0.00	0.20	0.00	0.00	0.00	Bacteria	Proteobacteria	Gammaproteobacteria	Alteromonadales	Alteromonadaceae	Haliea
0.00	0.00	0.00	0.00	0.15	0.00	0.00	0.00	Bacteria	Proteobacteria	Gammaproteobacteria	Alteromonadales	Alteromonadaceae	OM60(NOR5) clade
0.00	0.00	0.00	0.00	0.02	0.00	0.00	0.00	Bacteria	Proteobacteria	Gammaproteobacteria	Alteromonadales	Shewanellaceae	Shewanella
0.00	0.00	0.00	0.00	0.05	0.00	0.00	0.00	Bacteria	Proteobacteria	Gammaproteobacteria	Chromatiales	Chromatiaceae	Nitrosococcus
0.00	0.00	0.01	0.00	0.00	0.00	0.01	0.00	Bacteria	Proteobacteria	Gammaproteobacteria	Chromatiales	Chromatiaceae	Rheinheimera
0.00	0.00	0.00	0.00	0.05	0.00	0.00	0.00	Bacteria	Proteobacteria	Gammaproteobacteria	Chromatiales	Ectothiorhodospiraceae	Acidiferrobacter
0.86	0.00	0.00	0.00	0.00	0.00	0.00	0.00	Bacteria	Proteobacteria	Gammaproteobacteria	Chromatiales	Halothiobacillaceae	Halothiobacillus
0.43	0.00	0.00	0.00	0.00	0.00	0.00	0.00	Bacteria	Proteobacteria	Gammaproteobacteria	Chromatiales	Halothiobacillaceae	uncultured
0.00	0.00	0.00	0.00	0.00	0.00	0.00	0.01	Bacteria	Proteobacteria	Gammaproteobacteria	Enterobacteriales	Enterobacteriaceae	Enterobacter
0.00	0.00	0.00	0.00	0.05	0.02	0.00	0.00	Bacteria	Proteobacteria	Gammaproteobacteria	KI89A clade		
0.00	0.00	0.00	0.00	0.15	0.00	0.00	0.00	Bacteria	Proteobacteria	Gammaproteobacteria	Legionellales	Coxiellaceae	Coxiella
0.00	0.00	0.00	0.00	0.25	0.07	0.02	0.12	Bacteria	Proteobacteria	Gammaproteobacteria	Legionellales	Legionellaceae	Legionella
0.00	0.00	0.00	0.00	0.00	0.01	0.00	0.02	Bacteria	Proteobacteria	Gammaproteobacteria	Methylococcales	CABC2E06	
0.00	0.00	0.00	0.00	0.32	0.00	0.00	0.01	Bacteria	Proteobacteria	Gammaproteobacteria	Methylococcales	Crenotrichaceae	Crenothrix
0.43	0.10	0.00	0.01	0.20	0.38	0.00	0.32	Bacteria	Proteobacteria	Gammaproteobacteria	Methylococcales	Methylococcaceae	Methylobacter
0.00	0.00	0.00	0.00	0.02	0.00	0.00	0.00	Bacteria	Proteobacteria	Gammaproteobacteria	NKB5		
0.00	0.10	0.00	0.00	0.10	0.14	0.00	0.24	Bacteria	Proteobacteria	Gammaproteobacteria	Oceanospirillales	Oceanospirillaceae	Pseudospirillum
0.00	0.15	0.00	0.00	0.00	0.08	0.00	0.09	Bacteria	Proteobacteria	Gammaproteobacteria	Oceanospirillales	SAR86 clade	
0.00	0.00	0.00	0.00	0.02	0.00	0.00	0.00	Bacteria	Proteobacteria	Gammaproteobacteria	Oceanospirillales	oc58	
0.00	0.00	0.00	0.00	0.20	0.00	0.00	0.00	Bacteria	Proteobacteria	Gammaproteobacteria	Order Incertae Sedis	Family Incertae Sedis	Arenicella
0.00	0.00	0.00	0.00	0.05	0.00	0.00	0.00	Bacteria	Proteobacteria	Gammaproteobacteria	Order Incertae Sedis	Family Incertae Sedis	Marinicella
0.00	0.00	0.00	0.00	0.02	0.00	0.00	0.02	Bacteria	Proteobacteria	Gammaproteobacteria	Pseudomonadales	Moraxellaceae	Acinetobacter
0.00	0.00	0.00	0.00	0.00	0.00	0.00	0.01	Bacteria	Proteobacteria	Gammaproteobacteria	Pseudomonadales	Moraxellaceae	Perlucidibaca
0.00	0.00	0.00	0.00	0.02	0.00	0.00	0.00	Bacteria	Proteobacteria	Gammaproteobacteria	Pseudomonadales	Pseudomonadaceae	Cellvibrio
0.00	0.00	0.13	0.18	0.10	0.01	0.03	0.03	Bacteria	Proteobacteria	Gammaproteobacteria	Pseudomonadales	Pseudomonadaceae	Pseudomonas
0.00	0.05	0.33	0.66	0.00	0.00	0.23	0.07	Bacteria	Proteobacteria	Gammaproteobacteria	Pseudomonadales	Pseudomonadaceae	uncultured
0.00	0.00	0.00	0.00	0.00	0.01	0.00	0.01	Bacteria	Proteobacteria	Gammaproteobacteria	Thiotrichales	Piscirickettsiaceae	Piscirickettsia
0.00	0.00	0.00	0.00	0.10	0.00	0.00	0.01	Bacteria	Proteobacteria	Gammaproteobacteria	Thiotrichales	Thiotrichaceae	Thiothrix
0.00	0.00	0.00	0.00	0.00	0.00	0.00	0.03	Bacteria	Proteobacteria	Gammaproteobacteria	Xanthomonadales	Sinobacteraceae	Hydrocarboniphaga
0.00	0.00	0.00	0.00	0.02	0.00	0.00	0.00	Bacteria	Proteobacteria	Gammaproteobacteria	Xanthomonadales	Sinobacteraceae	JTB255 marine benthic group
0.00	0.00	0.00	0.00	0.00	0.00	0.00	0.04	Bacteria	Proteobacteria	Gammaproteobacteria	Xanthomonadales	Sinobacteraceae	Nevskia

Phase(I)-EL-PA	Phase(I)-EL-FL	Phase(II)-EL-PA	Phase(II)-EL-FL	Phase(I)-HL-PA	Phase(I)-HL-FL	Phase(II)-HL-PA	Phase(II)-HL-FL	Domain	Phylum	Class	Order	Family	Genus
0.00	0.00	0.00	0.00	0.00	0.00	0.00	0.01	Bacteria	Proteobacteria	Gammaproteobacteria	Xanthomonadales	Sinobacteraceae	Solimonas
0.00	0.00	0.00	0.00	0.02	0.00	0.00	0.00	Bacteria	Proteobacteria	Gammaproteobacteria	Xanthomonadales	Sinobacteraceae	Steroidobacter
0.00	0.07	0.01	0.00	1.62	0.05	0.02	0.11	Bacteria	Proteobacteria	Gammaproteobacteria	Xanthomonadales	Sinobacteraceae	uncultured
0.00	0.00	0.00	0.00	0.62	0.00	0.00	0.00	Bacteria	Proteobacteria	Gammaproteobacteria	Xanthomonadales	Xanthomonadaceae	Arenimonas
0.43	0.00	0.01	0.00	0.00	0.00	0.00	0.00	Bacteria	Proteobacteria	Gammaproteobacteria	Xanthomonadales	Xanthomonadaceae	Luteimonas
0.00	0.00	0.00	0.00	0.00	0.00	0.00	0.00	Bacteria	Proteobacteria	Gammaproteobacteria	Xanthomonadales	Xanthomonadaceae	Lysobacter
0.43	0.00	0.00	0.00	0.05	0.00	0.00	0.00	Bacteria	Proteobacteria	Gammaproteobacteria	Xanthomonadales	Xanthomonadaceae	Pseudofulvimonas
0.00	0.00	0.00	0.00	0.02	0.00	0.00	0.00	Bacteria	Proteobacteria	Gammaproteobacteria	Xanthomonadales	Xanthomonadaceae	Silanimonas
0.00	0.00	0.00	0.00	0.00	0.00	0.00	0.01	Bacteria	Proteobacteria	Gammaproteobacteria	Xanthomonadales	Xanthomonadaceae	Stenotrophomonas
0.00	0.00	0.00	0.00	0.12	0.02	0.02	0.01	Bacteria	Proteobacteria	Gammaproteobacteria	Xanthomonadales	Xanthomonadaceae	Thermomonas
0.00	0.00	0.00	0.00	0.35	0.00	0.00	0.00	Bacteria	Proteobacteria	Gammaproteobacteria	Xanthomonadales	Xanthomonadaceae	uncultured
0.00	0.00	0.00	0.00	0.00	0.00	0.00	0.01	Bacteria	Proteobacteria	MACA-EFT26			
0.00	0.00	0.00	0.00	0.15	0.01	0.01	0.01	Bacteria	Proteobacteria	SPOTSOCT00m83			
0.00	0.00	0.00	0.00	0.05	0.01	0.00	0.00	Bacteria	Proteobacteria	TA18			
0.00	0.00	0.00	0.00	0.05	0.00	0.00	0.00	Bacteria	Spirochaetes	Spirochaetes	Kazan-3B-09		
0.00	0.00	0.00	0.00	0.00	0.02	0.00	0.01	Bacteria	Spirochaetes	Spirochaetes	Spirochaetales	Leptospiraceae	Leptospira
0.00	0.00	0.00	0.00	0.82	0.02	0.00	0.00	Bacteria	Spirochaetes	Spirochaetes	Spirochaetales	Spirochaetaceae	Spirochaeta
0.00	0.00	0.00	0.00	0.12	0.00	0.00	0.00	Bacteria	Spirochaetes	Spirochaetes	Spirochaetales	Spirochaetaceae	uncultured
0.00	0.00	0.00	0.00	0.47	0.00	0.00	0.00	Bacteria	TA06				
0.00	0.00	0.00	0.00	0.10	0.01	0.00	0.00	Bacteria	TM6				
0.00	0.00	0.00	0.00	0.00	0.00	0.00	0.00	Bacteria	Tenericutes	Mollicutes	Entomoplasmatales	Entomoplasmataceae	Entomoplasma
0.00	0.00	0.00	0.00	0.00	0.01	0.01	0.01	Bacteria	Tenericutes	Mollicutes	Entomoplasmatales	Spiroplasmataceae	Spiroplasma
0.00	0.00	0.00	0.00	0.00	0.00	0.00	0.01	Bacteria	Tenericutes	Mollicutes	RF9		
0.00	0.00	0.00	0.00	0.00	0.00	0.00	0.01	Bacteria	Verrucomicrobia	Candidatus Methylacidiphilum			
0.00	0.00	0.00	0.00	0.42	0.03	0.01	0.06	Bacteria	Verrucomicrobia	OPB35 soil group			
0.00	0.00	0.00	0.00	0.00	0.00	0.00	0.03	Bacteria	Verrucomicrobia	OPB35 soil group	Pedosphaera		
0.43	0.51	0.00	0.00	0.45	1.38	0.01	1.70	Bacteria	Verrucomicrobia	Opitutae	Opitutales	Opitutaceae	Opitutus
1.29	4.47	0.04	0.02	1.49	2.05	0.01	1.54	Bacteria	Verrucomicrobia	Opitutae	vadinHA64		
0.00	0.00	0.00	0.00	0.07	0.00	0.00	0.00	Bacteria	Verrucomicrobia	S-BQ2-57 soil group			
0.00	0.00	0.00	0.00	0.32	0.00	0.01	0.01	Bacteria	Verrucomicrobia	Spartobacteria	Chthoniobacterales	Chthoniobacteraceae	Chthoniobacter
0.00	0.00	0.00	0.00	0.05	0.00	0.00	0.00	Bacteria	Verrucomicrobia	Spartobacteria	Chthoniobacterales	DA101 soil group	
0.00	0.00	0.00	0.00	0.00	0.00	0.00	0.01	Bacteria	Verrucomicrobia	Spartobacteria	Chthoniobacterales	FukuN18 freshwater group	
0.00	0.02	0.00	0.00	0.00	0.00	0.00	0.00	Bacteria	Verrucomicrobia	Spartobacteria	Chthoniobacterales	LD29	
0.00	0.00	0.01	0.00	0.00	0.00	0.00	0.00	Bacteria	Verrucomicrobia	Verrucomicrobiae	Verrucomicrobiales	Verrucomicrobiaceae	Haloferula
0.00	0.00	0.00	0.00	0.02	0.00	0.00	0.00	Bacteria	Verrucomicrobia	Verrucomicrobiae	Verrucomicrobiales	Verrucomicrobiaceae	Prosthecobacter
0.00	0.00	0.00	0.00	0.17	0.01	0.01	0.01	Bacteria	Verrucomicrobia	Verrucomicrobiae	Verrucomicrobiales	Verrucomicrobiaceae	uncultured
0.00	0.05	0.00	0.00	0.35	0.13	0.02	0.10	Bacteria	WCHB1-60				
11.59	0.90	0.65	0.24	5.55	1.35	0.47	2.62	No Relative					
