# Supplementary material for: Massive Regime Shifts and High Activity of Heterotrophic Bacteria in an Ice-Covered Lake
Source: PLoS One. 2014 Nov 24;9(11):e113611. doi: 10.1371/journal.pone.0113611 (PMC4242651; doi:10.1371/journal.pone.0113611)
Supplement: Table S5 — Under-ice phytoplankton blooms in limnic and marine systems. (DOC) [file pone.0113611.s009.doc]

Table S5 Under-ice phytoplankton blooms in limnic and marine systems

| **Dominant phytoplankton** | **Sampling year** | **Place observed** | **Reference** |
| --- | --- | --- | --- |
| **Limnic Environments** | | | |
| Diatoms  Golden algae  Dinoflagellates | 1977-1978 | White Heron Lake, Pennsylvania U.S.A. | [1] |
| Diatoms  Cyanobacteria | 2005 | Lac Saint Pierre of St. Lawrence River Canada | [2] |
| Diatoms  Golden algae  Dinoflagellates  Green algae | 1962-1963 | Black Lake Colorado, Pass Lake Ontario, Tea Lake Ontario | [3] |
| Diatoms  Golden algae  Green algae  Cryptomonads | 2005  1963 | Lake Päijänne, Finland | [4,5] |
| Golden algae  Green algae | 1969-1972 | Heywood Lake Antarctica | [6] |
| Diatoms  Golden algae  Dinoflagellates  Cryptomonads | 1961-1962 | Beaver Pond, Massachusetts, U.S.A | [7] |
| Green algae  Cryptomonads  Cyanobacteria | 1990, 1991 | Lake Opinicon, Canada  Upper Rock Lake, Canada | [8] |
| Golden algae  Green algae | Lake Rédo 1992, 1994;  Schwarzsee ob Sölden 1994; Gossenköllesee 1994 | Lake Redó, Spain,  Schwarzsee ob Sölden, Austria  Gossenköllesee, Austria | [9] |
| Diatoms  Green algae  Cryptomonads  Cyanobacteria | 1968 - present | Neusiedler See, Austria | [10] |
| Diatoms  Golden algae | 2004 | Lake Pääjärvi, Finland | [11] |
| Golden algae | 1974 | Lago Santo Parmense, Italy | [12] |
| Golden algae | 1962-1963 | Lake in the English Lake District, UK | [13] |
| Diatoms | 1994 | Lake Baikal, Russia | [14,15] |
| Diatoms | 1967-1971 | Lake Haruna, Japap | [16] |
| Dinoflagellates |  | Lapland | [17] |
| Cyanobacteria  Dinoflagellates | During the two austral summers | Antarctic lakes (Vanda and Bonney) | [18] |
| Cyanobacteria | 2009-2010 | Lake Stechlin | [19] and this study |
| **Marine Environments** | | | |
| Diatoms | 2012 | Arctic Sea | [20] |
| Diatoms |  | Arctic Sea | [21] |
| Diatoms | June 1998 | Chukchi sea (Arctic) | [22] |
| Diatoms | June 1993 | Northeast Greenland | [23] |
| Diatoms | 1998 | Ice camp drift in the Canadian Basin (Arctic) | [24] |

1. Campbell JM, Haase BL (1981) Availability of suitable phytoplanktonic food for zooplankton in an ice-covered lake. Hydrobiologia 79: 113–119. doi:10.1007/BF00006117.

2. Frenette J-J, Thibeault P, Lapierre J-F, Hamilton PB (2008) Presence of Algae in Freshwater Ice Cover of Fluvial Lac Saint-Pierre (St. Lawrence River, Canada). J Phycol 44: 284–291. doi:10.1111/j.1529-8817.2008.00481.x.

3. Pennak R (1968) Field and experimental winter limnology of three Colorado mountain lakes. Ecology 49: 505–520.

4. Kiili M, Pulkkanen M, Salonen K (2009) Distribution and development of under-ice phytoplankton in 90-m deep water column of Lake Päijänne (Finland) during spring convection. Aquat Ecol 43: 707–713. doi:10.1007/s10452-009-9262-7.

5. Granberg K (1973) The eutrophication and pollution of Lake Paijanne, Central Finland. Helsinki.

6. Light J, Ellis-Evans J, Priddle J (1981) Phytoplankton ecology in an Antarctic lake. Freshw Biol 11: 11–26.

7. Wright R (1964) Dynamics of a phytoplankton community in an ice-covered lake. Limnol Oceanogr 9: 163–178.

8. Agbeti M, Smol J (1995) Chrysophyte population and encystment patterns in two Canadian lakes. J Phycol 31: 70–78. doi:10.1111/j.0022-3646.1995.00070.x.

9. Felip M, Sattler B, Psenner R, Catalan J (1995) Highly active microbial communities in the ice and snow cover of high mountain lakes. Appl Environ Microbiol 61: 2394.

10. Dokulil MT, Herzig A (2009) An analysis of long-term winter data on phytoplankton and zooplankton in Neusiedler See, a shallow temperate lake, Austria. Aquat Ecol 43: 715–725. doi:10.1007/s10452-009-9282-3.

11. Vehmaa A, Salonen K (2009) Development of phytoplankton in Lake Pääjärvi (Finland) during under-ice convective mixing period. Aquat Ecol 43: 693–705. doi:10.1007/s10452-009-9273-4.

12. Ferrari I (1976) Winter limnology of a mountain lake: Lago Santo Parmense (northern Appennines, Italy). Hydrobiologia 51: 245–257.

13. Swale E, Belcher J (1966) Ochromonas ostreaeformis nov. sp., a large compressed chrysomonad. New Phytol 65: 267–272.

14. Sigee DC (2005) Biodiversity and dynamic interactions of microorganisms in the aquatic environments. Freshwater microbiology. Vol. 2.

15. Jewson DH, Granin NG, Zhdanov A a., Gnatovsky RY (2009) Effect of snow depth on under-ice irradiance and growth of Aulacoseira baicalensis in Lake Baikal. Aquat Ecol 43: 673–679. doi:10.1007/s10452-009-9267-2.

16. Maeda O, Ichimura S (1973) On the high density of a phytoplankton population found in a lake under ice. Int Rev der gesamten Hydrobiol und Hydrogr 58: 673–685.

17. Rodhe W (1955) Can plankton production proceed during winter darkness in subarctic lakes. Verh Int Ver Limnol 12: 117–122.

18. Goldman C, Mason D, Hobbie J (1967) Two Antarctic desert lakes. Limnol Oceanogr 12: 295–310.

19. Üveges V, Tapolczai K, Krienitz L, Padisák J (2012) Photosynthetic characteristics and physiological plasticity of an Aphanizomenon flos-aquae (Cyanobacteria, Nostocaceae) winter bloom in a deep oligo-mesotrophic lake (Lake Stechlin, Germany). Hydrobiologia 698: 263–272. doi:10.1007/s10750-012-1103-3.

20. Boetius A, Albrecht S, Bakker K, Bienhold C, Felden J, et al. (2013) Export of algal biomass from the melting Arctic sea ice. Science 339: 1430–1432. doi:10.1126/science.1231346.

21. Arrigo KR, Perovich DK, Pickart RS, Brown ZW, van Dijken GL, et al. (2012) Massive phytoplankton blooms under Arctic sea ice. Science 336: 1408. doi:10.1126/science.1215065.

22. Ambrose WG, Quillfeldt C Von, Clough LM, Tilney PVR, Tucker T (2005) The sub-ice algal community in the Chukchi sea: large- and small-scale patterns of abundance based on images from a remotely operated vehicle. Polar Biol 28: 784–795. doi:10.1007/s00300-005-0002-8.

23. Gutt J (1995) The occurrence of sub-ice algal aggregations off northeast Greenland. 119: 247–252.

24. Melnikov IA, Kolosova EG, Welch HE, Zhitina LS (2002) Sea ice biological communities and nutrient dynamics in the Canada Basin of the Arctic Ocean. Deep Sea Res Part I Oceanogr Res Pap 49: 1623–1649. doi:10.1016/S0967-0637(02)00042-0.
